# Mild hyperthermia enhanced synergistic uric acid degradation and multiple ROS elimination for an effective acute gout therapy

**DOI:** 10.1186/s12951-024-02539-9

**Published:** 2024-05-22

**Authors:** Pei Zhao, Hua-Zhong Hu, Xiao-Tong Chen, Qi-Yun Jiang, Xue-Zhao Yu, Xiao-Lin Cen, Shi-Qing Lin, Sui-qing Mai, Wei-lin Pang, Jin-Xiang Chen, Qun Zhang

**Affiliations:** 1 Guangdong Provincial Key Laboratory of Bone and Joint Degeneration Diseases, Office of Clinical Trial of Drug, The Third Affiliated Hospital, Southern Medical University, Guangzhou, 510663 Guangdong China; 2 NMPA Key Laboratory for Research and Evaluation of Drug Metabolism, Guangdong Provincial Key Laboratory of New Drug Screening, School of Pharmaceutical Sciences, Southern Medical University, Guangzhou, 510515 Guangdong China; 3 School of Chinese Medicine, Southern Medical University, Guangzhou, 510515 China

**Keywords:** Gouty microenvironment, Uric acid degradation, Anti-inflammatory effects, Reactive oxygen species (ROS) scavenging, Photothermal therapy

## Abstract

**Background:**

Acute gouty is caused by the excessive accumulation of Monosodium Urate (MSU) crystals within various parts of the body, which leads to a deterioration of the local microenvironment. This degradation is marked by elevated levels of uric acid (UA), increased reactive oxygen species (ROS) production, hypoxic conditions, an upsurge in pro-inflammatory mediators, and mitochondrial dysfunction.

**Results:**

In this study, we developed a multifunctional nanoparticle of polydopamine-platinum (PDA@Pt) to combat acute gout by leveraging mild hyperthermia to synergistically enhance UA degradation and anti-inflammatory effect. Herein, PDA acts as a foundational template that facilitates the growth of a Pt shell on the surface of its nanospheres, leading to the formation of the PDA@Pt nanomedicine. Within this therapeutic agent, the Pt nanoparticle catalyzes the decomposition of UA and actively breaks down endogenous hydrogen peroxide (H_2_O_2_) to produce O_2_, which helps to alleviate hypoxic conditions. Concurrently, the PDA component possesses exceptional capacity for ROS scavenging. Most significantly, Both PDA and Pt shell exhibit absorption in the Near-Infrared-II (NIR-II) region, which not only endow PDA@Pt with superior photothermal conversion efficiency for effective photothermal therapy (PTT) but also substantially enhances the nanomedicine’s capacity for UA degradation, O_2_ production and ROS scavenging enzymatic activities. This photothermally-enhanced approach effectively facilitates the repair of mitochondrial damage and downregulates the NF-κB signaling pathway to inhibit the expression of pro-inflammatory cytokines.

**Conclusions:**

The multifunctional nanomedicine PDA@Pt exhibits exceptional efficacy in UA reduction and anti-inflammatory effects, presenting a promising potential therapeutic strategy for the management of acute gout.

**Graphical Abstract:**

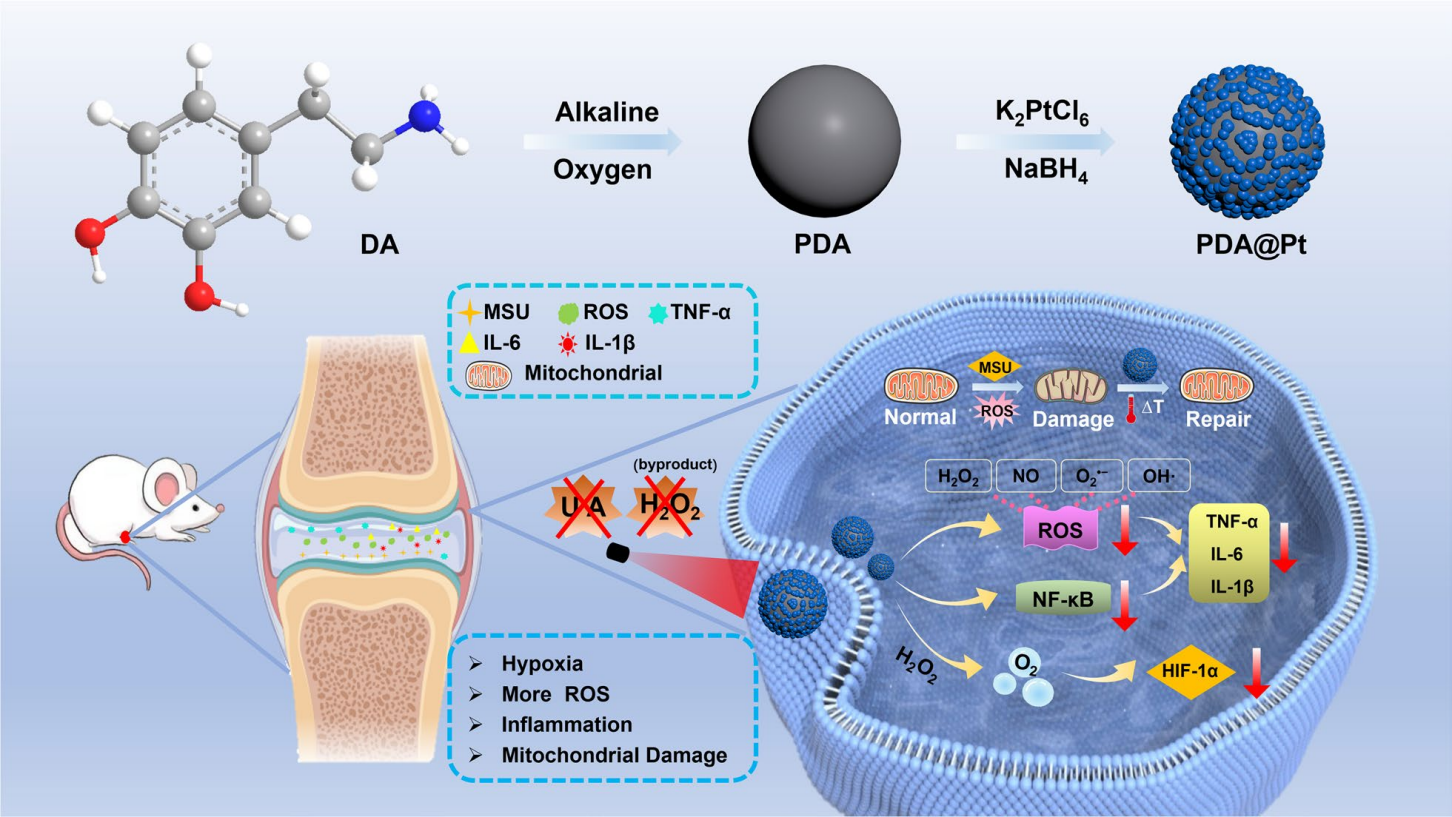

**Supplementary Information:**

The online version contains supplementary material available at 10.1186/s12951-024-02539-9.

## Introduction

Acute gout is a result of the accumulation of Monosodium Urate (MSU) in various parts of the body, including joints, bones, cartilage, and other tissues [1]. This leads to symptoms such as joint pain, stiffness, warmth, redness, and limited mobility, which can significantly impact the quality of patient’s life [2]. The pathological basis of acute gout is related to the deficiency of uricase to break down the uric acid (UA), leading to a continuous increase in UA concentration within tissues until it reaches saturation and forms MSU crystals [3, 4]. These formed MSU crystals can stimulate the release of various inflammatory factors, including TNF-α, IL-6, and IL-1β, which is achieved by activating the NF-κB signaling pathway [5]. Furthermore, the deposition of MSU crystals in joints can trigger oxidative stress, which generates a substantial amount of reactive oxygen species (ROS), particularly H_2_O_2_. Oxidative stress can cause abnormal expression of various inflammatory markers, resulting in mitochondrial dysfunction and aberrant cellular energy metabolism, further increasing the joint burden and exacerbating joint damage [6, 7].

The clinical treatment of acute gout primarily involves surgical and pharmaceutical interventions. While surgical intervention may offer more rapid relief, it is associated with risks such as significant injury, potential infections, a longer recovery time, and higher costs. On the other hand, drug treatments for gout primarily focus on anti-inflammatory measures and management of UA levels. Common anti-inflammatory medications include non-steroidal anti-inflammatory drugs (NSAIDs), systemic corticosteroids, and colchicine. Furthermore, the IL-1 receptor antagonist Lesinurad is effective in reducing inflammation. However, the bioavailability of these drugs is limited, leading to various adverse reactions [8–11]. UA-targeted drugs typically aim to inhibit its synthesis, promote its excretion, or involve uricase preparations. Uricase can catalyze the degradation of UA into more soluble compounds, providing fast and significant effects for gout treatment [12]. However, while uricase can effectively excrete UA out of the body in a short period, some patients may experience allergic symptoms such as red rashes and itching after using it. These reactions can be life-threatening in severe cases. Additionally, uricase can cause gastrointestinal discomfort, including nausea and vomiting, leukopenia, hair loss, and other adverse reactions. Currently, Rasburicase and Pegloticase are being studied in relation to these potential side effects [13, 14]. As acute gout progresses, uricase catalyzes the breakdown of UA, which generates a multitude of ROS, particularly hydrogen peroxide (H_2_O_2_) [15]. These ROS aggravate an inflammatory response in acute gout lesions, and the utilization of uricase treatment may further exacerbate joint damage.

Recently, there has been a surge of interest in artificial nanozymes. These enzymatic nanoparticles can mimic the functions of natural enzymes and possess qualities such as stability, minimal toxicity, low cost, and tunability [16]. Current nanozymes can be broadly categorized into three groups: (1) metal-based nanoparticles, such as platinum (Pt) and palladium (Pd); (2) metal oxide-based nanoparticles, such as CeO_2_ and Fe_3_O_4_; and (3) carbon nanostructures, which encompass carbon dots and carbon tubes [17]. Among these nanozymes, Pt nanoparticles have demonstrated great potential due to their inherent multiple enzyme activities, which include uricase activity, peroxidase (POD) activity, catalase (CAT) activity, and superoxide dismutase (SOD) activity [18, 19]. The enzymatic activity of Pt nanoparticles is directly proportional to their size, which increases as their particle size decreases. However, smaller nanoparticles are prone to agglomeration and rapid excretion through the kidneys, evading liver uptake and reducing the circulation time in the body, ultimately diminishing the therapeutic efficacy [20].

A variety of nanoparticles, including metal–organic frameworks (MOFs), silicon dioxide (SiO_2_), polydopamine (PDA), and many others, have been employed as templates for the synthesis of smaller nanozymes [21–23]. Among those various templates, PDA has attracted immense interest owing to its high surface area and abundance of amino functional groups, which offer numerous binding sites for metal ions and facilitate nanozyme growth. Additionally, PDA exhibits remarkable ROS scavenging ability and holds great potential in photothermal therapeutic applications. The photothermal effect can alleviate inflammation and significantly boost nanozyme biological activity [24]. In recent years, photothermal therapy (PTT) has become a promising treatment modality, attributed to its low toxicity and efficient photothermal conversion properties [25]. As opposed to conventional photothermal therapy, the application of gentle photothermal therapy with controlled temperature can prevent significant tissue damage and the formation of unsightly scars [26, 27].

**Scheme 1.**
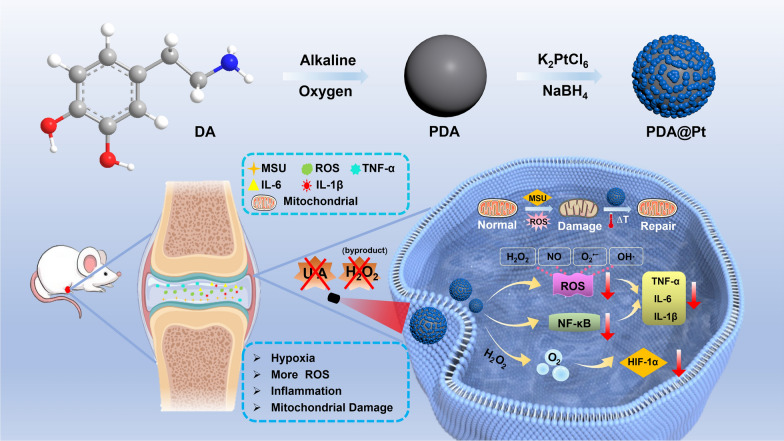
Schematic diagram of the preparation process of PDA@Pt nanomedicine and its in vivo mechanism for treating acute gout

In this study, we have developed a core–shell structure, namely polydopamine-platinum (PDA@Pt) nanozyme for the treatment of acute gouty (Scheme 1). The PDA nanoparticles are formed through the oxidative polymerization of dopamine in alkaline conditions, resulting in regular spherical nanoparticles. The large surface of PDA is rich in amino functional groups, which enable the coordination of Pt ions with these groups. The nucleation of Pt nanoparticles occurs through the reduction of Pt ions by NaBH_4,_ and further growth of Pt nanoshell. In the microenvironment of acute gout, where UA and H_2_O_2_ levels are elevated, the nano-Pt in PDA@Pt not only catalyzes the degradation of UA through its uricase activity but also decomposes H_2_O_2_ through its CAT activity, producing oxygen (O_2_) and alleviating cellular hypoxia. This process helps to relieve the symptoms of acute gout. Additionally, both PDA and nano-Pt exhibit the powerful SOD activity for the elimination of ROS, which can effectively inhibit the progression of the inflammatory state. Most importantly, the photothermal properties of PDA and Pt-shell enhance the enzymatic activities in PDA@Pt, further amplifying its therapeutic potential. When combined with an NIR laser (808 nm), PDA@Pt can prevent the activation of the NF-κB signaling pathway and down-regulate the release of inflammatory cytokines. Additionally, PDA@Pt repairs mitochondrial damage, regulates intracellular ATP content and calcium homeostasis to protect mitochondrial function, and maintains the normal physiological function of cells. This nanosystem enhances the solubility of MSU with the aid of an NIR laser, indirectly promoting the metabolic decomposition of UA. Compared to other nanoparticle formulations, this designed nanosystem offers advantages such as broad enzyme activity, fewer by-products, and simpler synthesis process. In an acute gout model induced by MSU in rats, the developed nanosystem significantly ameliorated the inflammatory response in the joints. This finding highlights the potential of PDA@Pt as a promising therapeutic agent for the treatment of gout and related inflammatory diseases.

## Methods

### Materials

Potassium chloroplatinate (K_2_PtCl_6_), sodium borohydride (NaBH_4_), and dopamine hydrochloride (DA) were purchased from Aladdin (Shanghai, China). Lipopolysaccharides (LPS), 4′6-diamidino-2-phenylindole (DAPI), and 3-(4,5-dimethylthiazol-2-yl)-2,5-diphenyltetrazolium bromide (MTT) were purchased from Sigma-Aldrich. Uric acid (UA), uricase, and potassium iodide (KI) were all procured from Macklin. Hydrogen peroxide (H_2_O_2_) was sourced from Guangzhou Chemical Reagent Factory. ATP Assay Kit, Calcium Ion Fluorescent Probe (Fluo-4 AM), 2, 7-dichlorodihydrofluorescein diacetate (DCFH-DA) and live (calcein-AM)/dead (propidium iodide, PI) dyes were purchased from Beyotime Institute of Biotechnology (China). The Dihydroethidium (DHE) fluorescent probe was purchased from Yisheng Biotechnology (Shanghai) Co., Ltd. The NO (DAF-FM) fluorescent probe was acquired from AAT Bioquest Inc. Tetramethylrhodamine ethyl ester (TMRE) was purchased from Beijing Coolaibo Technology Co., Ltd.

### Measurements and characterizations

The surface charge (zeta potential) of the different nanoparticles was analyzed using a Malvern Zetasizer Nano ZS (Malvern, England). A Perkin Elmer LAMBDA 25 spectrometer was used in detecting the ultraviolet–visible (UV–Vis) absorption spectra. The powder X-ray diffraction (PXRD) patterns were measured with the X’Pert-Pro MPD diffractometer (Netherlands PANalytical). The X-ray photoelectron spectroscopy (XPS) was recorded on a Thermo Fisher Scientific Nexsa spectrometer. The morphologies and structures of different nanomaterials were characterized through a transmission electron microscope (TEM, H—7650, Hitachi, Japan). Agilent 8900 inductively coupled plasma-mass spectrometry (ICP-MS) was used to measure the proportion of metal contents in PDA@Pt. O_2_ levels were detected using a dissolved oxygen meter (JPB-607A model portable dissolved oxygen meter). Illumination using an 808nm laser (Beijing Raycus Power, LWIRL808nm-30W-F). An infrared (IR) thermal image camera (FLIR E60, FLIR Systems, Inc., USA) was used to monitor the temperature changes. Confocal laser scanning microscope (CLSM) images were taken using ZEISS LSM 880.

### Preparation of PDA nanozymes

PDA was synthesized according to the previous literature [28]. First, ammonia water (2 mL), ethanol (40 mL), and deionized water (DI, 90 mL) were mixed and stirred for 30 min. Then, 10 mL DA solution (50 mg/mL) was introduced into the mixture. The reaction proceeded for 24 h with continuous stirring. Next, it was centrifuged at 13000 rpm for 10 min and washed three times with DI water to obtain PDA. The quantity was determined and stored at 4 °C.

### Preparation of PDA@Pt nanozymes

The obtained PDA solution (2 mg/mL, 1 mL) was introduced into a K_2_PtCl_6_ solution (0.6 mg/mL, 10 mL). The mixture was stirred continuously for 40 min at room temperature. Subsequently, a NaBH_4_ solution (0.1 mg/mL, 50 mL) was slowly added to the solution, and stirring was maintained for 20 min at room temperature. Finally, the resulting mixture was subjected to a centrifugation process (10,000 rpm, 5 min) to obtain PDA@Pt. Following this, the product was washed three times with DI water and stored at 4 °C.

### Preparation of MSU

1 g UA was dissolved in 200 mL DI water containing 6 mL sodium hydroxide (NaOH, 1M) at 70 °C. The pH value of the solution formed was adjusted to 7.1 ~ 7.2 using hydrochloric acid (HCl) or NaOH and then filtered the solution. The filtered solution was naturally cooled and slowly stirred at room temperature, then stored overnight at 4 °C. The next day, the precipitate was collected by centrifugation and dried under low heat.

### ICP-MS analysis

PDA@Pt (0.45 mg) was dissolved in 4 mL aqua regia for 24 h. Subsequently, take 2 mL from this solution and dilute it with 6 mL of deionized water for subsequent analysis by ICP-MS.

### Photothermal test

To evaluate the photothermal properties of PDA and PDA@Pt, the temperature differentials induced by NIR laser irradiation in diverse solutions were recorded every 30 s, and graphical representations were constructed based on the irradiation duration. The PDA (72 μg/mL, 1 mL) and PDA@Pt (which contains 72 μg/mL PDA, 100 μg/mL, 1 mL) solutions were prepared and subjected to irradiation with an 808 nm laser (1.0 W/cm^2^, 10 min) with concurrent monitoring of temperature changes. A uniform concentration of the PDA@Pt solution was exposed to an 808 nm laser at varying power (0.5, 1, 1.5, 2.0 W/cm^2^), with temperature changes being recorded in real-time. Additionally, the different concentrations of PDA@Pt solutions (0, 25, 50, 100 μg/mL) were irradiated using an 808 nm laser (1 W/cm^2^, 10 min), with concurrent monitoring of temperature variations. To assess the photothermal stability of the nanosystem, a solution of PDA@Pt (100 μg/mL) was exposed to 808 nm laser irradiation for 10 min and then allowed to cool to ambient temperature. This on/off cycle was repeated 5 times, with the temperature differentials of the solution continuously recorded throughout. The photothermal conversion efficiency (η) of PDA@Pt can be calculated using Eq. (1):

1 $$\eta \, = \, \left[ {{\text{hS }}\left( {{\text{T}}_{{{\text{max}}}} - {\text{T}}_{{{\text{surr}}}} } \right) - {\text{Q}}_{{{\text{dis}}}} } \right]/{\text{I }}\left( {{1} - {1}0^{{ - {\text{A}}}} } \right)$$

“h” represents the heat transfer coefficient, “S” denotes the surface area of the container, “T_max_” signifies the maximum temperature attained, “T_surr_” corresponds to the ambient temperature, “Q_dis_” accounts for heat dissipation due to light absorption by the container, “I” stands for the laser power density, and “A” represents the absorbance of PDA@Pt at 808 nm. The value of “hS” can be computed using Eq. (2).

2 $${\text{hS }} = {\text{ mC}}_{{{\text{water}}}} /\tau_{{\text{s}}}$$

“m” represents the mass of the solution being heated, “C_water_” signifies the specific heat capacity of water, and “τ_s_” is the time constant of the sample system. The time constant can be calculated using Eq. (3):

3 $$\tau_{{\text{s}}} = \, - {\text{ln}}\theta /{\text{t}}$$

Through the analysis of the sample's cooling curve, a standard curve is constructed correlating the cooling time “t” and “-lnθ”. The slope of the regression equation derived from this curve represents the time constant, denoted as “τ_s_”. The value of θ can be calculated using Eq. (4):

4 $$\theta \, = \, \left( {{\text{T}} - {\text{T}}_{{{\text{surr}}}} } \right)/\left( {{\text{T}}_{{{\text{max}}}} - {\text{T}}_{{{\text{surr}}}} } \right)$$

### ABTS^+•^ and DPPH^•^ scavenging activity

The Total Antioxidant Capacity Assay Kit (using the ABTS method) was employed to evaluate the inhibitory effect of PDA@Pt on ABTS^+•^. According to the provided instructions, the process was initiated by combining the ABTS solution with the oxidant at a 1:1 volume ratio to prepare the ABTS working stock solution. This solution was stored in darkness at room temperature overnight. On the subsequent day, the ABTS working stock solution was diluted 30–50 times with PBS to formulate the ABTS working solution. Various concentrations of PDA, PDA + Laser, PDA@Pt, and PDA@Pt + Laser were meticulously prepared in PBS to establish a concentration gradient. Then, the ABTS working solution (200 μL) was dispensed into each well of a 96-well plate, including wells designated as blank controls. The sample (10 μL) was swiftly introduced into each well and allowed to incubate at room temperature for 2–6 min. The absorbance at 734 nm was measured using a multifunctional microplate reader.

The process was initiated by preparing a 0.1 mM DPPH^•^ ethanol solution and storing it in darkness. In a 96-well plate, the DPPH^•^ ethanol solution (190 μL) and varying concentrations of PDA, PDA + Laser, PDA@Pt, and PDA@Pt + Laser (10 μL) were added into each well. The mixture was incubated in the dark with agitation for 10 min, and then the absorbance at 517 nm was measured using a multifunctional microplate reader.

### O_2_^•−^, H_2_O_2,_ and ^•^OH elimination capability

The ability of PDA@Pt to scavenge O_2_^•−^ was evaluated using the classic nitroblue tetrazolium (NBT) colorimetric method. Following the instructions, the NBT/enzyme working solution and the reaction initiation working solution were prepared. In a 96-well plate, the NBT/enzyme working solution (160 μL), the reaction initiation working solution (20 μL), and different concentrations of PDA, PDA + Laser, PDA@Pt, PDA@Pt + Laser solutions (20 μL) were added into each well. A blank control well was set up. The plate was incubated at 37 °C for 30 min, and then the absorbance at 560 nm was measured using a multifunctional microplate reader.

Solutions of PDA, PDA + Laser, PDA@Pt, and PDA@Pt + Laser were reacted at various concentrations with 10 mM H_2_O_2_ for 15 min, followed by centrifugation. The supernatant was combined with an equal volume of 1M KI and incubated in the dark for 5 min. Afterward, the UV–Vis absorption at 350 nm was measured to calculate the degradation rate.

Terephthalic acid (TA) was employed in the experiment to capture ^•^OH, forming the fluorescent compound TA-OH. This method was utilized to assess the capability of PDA@Pt to scavenge ^•^OH. TA-OH exhibited blue fluorescence upon excitation at a wavelength of 315 nm. The solution comprising TA (5 mM), H_2_O_2_ (10 mM), and varying concentrations of PDA and PDA@Pt was prepared in PBS. These solutions were incubated for 12 h, then their fluorescence was measured using a fluorospectrophotometer.

### ^•^OH production capability

PDA@Pt (750 μL), TMB (16 mM, 50 μL), and H_2_O_2_ (5 mM, 200 μL) were introduced into an Eppendorf tube. Subsequently, the mixture was incubated at 37 °C for 15 min, and the absorbance spectrum was recorded across the wavelength range of 350 ~ 750 nm.

### UA degradation

Diverse concentrations of PDA, PDA@Pt, PDA@Pt + Laser, and UA (100 μM), were added into Eppendorf tubes and incubated at 37 °C. The catalytic degradation of UA was monitored by recording the changes in absorbance at 290 nm every 10 min using a UV–Vis spectrophotometer.

### Accumulated H_2_O_2_ after UA degradation

During the catalytic degradation of UA (1 mM) facilitated by natural uricase (5 U/mL) and PDA@Pt (50 μg/mL), 1 mL samples were collected every 10 min. These samples were centrifuged (12000 rpm, 4 min) to separate the supernatant. The supernatant was reacted with an equal volume of KI (1 M) at room temperature for 10 min. Subsequently, the absorbance at 350 nm was measured using a UV–Vis spectrophotometer.

### Dissolution of MSU

In a PBS solution, MSU and PDA were added. This mixture was exposed to an 808 nm laser at a power density of 1 W/cm^2^. At predetermined intervals, the solution was collected and centrifuged to obtain the supernatant. The absorbance at 290 nm was measured using a UV spectrophotometer.

### O_2_ generation capability

In a PBS solution, H_2_O_2_ (10 mM) was added, along with different concentrations of PDA, PDA@Pt, and PDA@Pt + laser solutions, with plain PBS serving as the control. The generation of O_2_ in each solution was continuously monitored for 12 min using a portable dissolved oxygen meter (Model JPB-607A).

### Cell culture and viability assay

Raw 264.7 cells, originating from mouse macrophages, were maintained in a complete medium consisting of 90% high-glucose Dulbecco’s Modified Eagle Medium (DMEM) and 10% fetal bovine serum (FBS), at 37 °C in a 5% CO_2_ incubator. Cell passaging was performed as dictated by the cell growth status. The culture conditions for HUVEC and FLS cells were analogous to those for Raw 264.7 cells. Upon exposure to 500 ng/mL LPS for 24 h, Raw 264.7 cells were induced to differentiate into the inflammatory cells.

To evaluate the cytotoxicity of PDA, PDA@Pt, and PDA@Pt + laser, Raw 264.7 cells, LPS-induced Raw 264.7 cells, HUVEC cells, and FLS cells were plated at a density of 8 × 10^3^ cells per well in a 96-well plate and cultured for 24 h. The cells were incubated with varying concentrations of PDA and PDA@Pt for 24 h. For PTT, Raw 264.7 cells were induced with 500 ng/mL LPS for 24 h. The old culture medium was removed, and the cells were incubated with different concentrations of PDA@Pt for another 24 h. The medium was replaced with fresh complete medium, each well was exposed to an 808 nm laser (1 W/cm^2^, 5 min). After 12 h of culture, the medium was discarded, and 10 μL of MTT solution (5 mg/mL) was added to each well and incubated for 4 ~ 6 h. Then, 100 μL of dimethyl sulfoxide (DMSO) was added to dissolve the formazan crystals. The absorbance at 490 nm was measured using a multi-mode microplate reader.

Abnormal cell death, triggered by H_2_O_2_ resulting from the catalytic degradation of UA by natural uricase and PDA@Pt, was investigated using the same cytotoxicity assay protocol as mentioned above, with the UA concentration at 1 mM.

### Live/dead staining assay

Raw 264.7 cells were co-cultured with LPS at a density of 7 × 10^4^ cells per well in a 12-well plate for 24 h. The cells were incubated with PBS, PBS + Laser, 28.8 μg/mL PDA, 40 μg/mL PDA@Pt (which contains 28.8 μg/mL PDA), and 40 μg/mL PDA@Pt + Laser (which contains 28.8 μg/mL PDA, 1 W/cm^2^, 5 min) for 24 h. Finally, the cells were incubated with calcein acetoxymethyl ester (calcein AM) and propidium iodide (PI) at 37 °C for 30 min. Imaging was performed under a confocal microscope, with calcein AM (Excitation: 488 nm) and PI (Excitation: 561 nm).

### Hemolysis assay

Fresh blood samples were obtained from healthy rats via orbital puncture, followed by centrifugation at 3500 rpm for 15 min to separate red blood cells (RBCs). The RBCs were then washed with PBS. RBCs (20 μL) were incubated with 1% Triton X-100 PBS, and varying concentrations of PDA@Pt (0, 10, 20, 40, 80, 160, and 320 μg/mL) at 37 °C for 3 h. Then, the mixture was centrifuged at 3500 rpm for 10 min, the supernatant was collected, and the absorbance at 540 nm was measured to calculate the hemolysis rate.

### Intracellular ROS scavenging and oxygen production

#### DAF-FM DA probe for NO

Raw 264.7 cells were cultured at a density of 7 × 10^4^ cells per well in a 12-well plate for 24 h, and then incubated with PBS, PBS + Laser, 28.8 μg/mL PDA, 40 μg/mL PDA@Pt, and 40 μg/mL PDA@Pt + Laser (1 W/cm^2^, 5 min) for another 24 h. The cells were gently washed three times with PBS, followed by incubation in a serum-free medium containing 1 mM H_2_O_2_ for 30 min (with light exposure during this incubation). After washes with PBS for 3 times, cells were incubated with 1 μM DAF-FM DA probe for 20 ~ 30 min. After washing with PBS for 2 times, the cells were observed using a confocal microscope. The fluorescence intensity was quantified with Image J (Excitation: 488 nm).

#### DHE probe for O_2_^•−^

Raw 264.7 cells were treated as described above. After incubation with 10 μM DHE for 60 min, imaging was performed under a laser confocal microscope. (Excitation: 518 nm).

#### DCFH-DA probe for H_2_O_2_-induced ROS

Raw 264.7 cells were treated as described previously. After staining with 40 μM DCFH-DA for 15 min at 37 °C, cells were imaged under a Laser confocal microscope. (Excitation: 488 nm).

#### [Ru (DPP)_3_] Cl_2_ probe for O_2_

Raw 264.7 cells were seeded into a 12-well plate and subjected to hypoxic conditions with LPS induction for 24 h to promote differentiation into inflammatory cells. This was followed by treatment with 4 μM [Ru (DPP)_3_] Cl_2_ for 4 h. Thereafter, the cells were subjected to various treatments: PBS, PBS + Laser, 28.8 μg/mL PDA, 40 μg/mL PDA@Pt, and 40 μg/mL PDA@Pt + Laser (1 W/cm^2^, 5 min). The cells are incubated for a further 24 h. Finally, the cells were washed with PBS for 3 times and imaged using a Laser confocal microscope. (Excitation: 488 nm).

#### DCFH-DA probe for LPS-induced ROS

Raw 264.7 cells were seeded into a 12-well plate and stimulated with LPS for 24 h to differentiate into inflammatory cells. Subsequently, the cells were subjected to various treatments: PBS, PBS + Laser, 28.8 μg/mL PDA, 40 μg/mL PDA@Pt, and 40 μg/mL PDA@Pt + Laser (1 W/cm^2^, 5 min). The cells were incubated for an additional 24 h. Afterward, the cells were gently washed with PBS for 3 times, followed by staining with 40 μM DCFH-DA for 15 min at 37 °C. Finally, the cells were washed with PBS for 3 times and imaged using a laser confocal microscope (Excitation: 488 nm).

#### ATP assay

For the LPS-induced Raw 264.7 cells, treatment was carried out following the previously outlined protocol. After pre-treatment of the cells, the intracellular ATP levels were determined using the Enhanced ATP Assay Kit (Beyotime, China). The ATP production was measured using a multi-function microplate reader in each group of cells.

#### Cytoplasmic Ca^2+^ concentration detection

Intracellular Ca^2+^ levels were determined using the Fluo-4 AM probe. LPS-induced Raw 264.7 cells were treated according to the protocol mentioned above. After washing, the cells were incubated with Fluo-4 AM (2 μM) at 37 °C for 30 min. After rinsing with PBS, the cells were incubated with DAPI (10 μg/mL) at 37 °C for 10 min. The cells were observed under an LSM 880 microscope. (Excitation for DAPI: 405 nm, Excitation for Fluo-4 AM: 488 nm).

#### Mitochondrial membrane potential detection

The treatment was conducted by the protocol for LPS-induced Raw 264.7 cells. After washing, the cells were incubated with 20 nM Tetramethylrhodamine Ethyl Ester (TMRE) at 37 °C for 30 min. Post PBS wash, the cells were incubated with DAPI (10 μg/mL) at 37 °C for 10 min. Observations were conducted using an LSM 880 microscope (Excitation for DAPI: 405 nm, Excitation for TMRE: 561 nm).

#### Immunofluorescent Staining Analysis:

Raw 264.7 cells were plated in confocal dishes and stimulated with LPS for 24 h. The cells were subjected to various treatments for 24 h [PBS, PBS + Laser, 28.8 μg/mL PDA, 40 μg/mL PDA@Pt, and 40 μg/mL PDA@Pt + Laser (1 W/cm^2^, 5 min)]. For HIF-1α IF staining, the cells were cultured under hypoxic conditions before staining. After washing three times with PBS, cells were fixed with 1 mL 4% paraformaldehyde for 15 min, permeabilized with 1 mL 0.1% Triton X-100 for 10 min at room temperature, and blocked with 1 mL 5% BSA for 30 min. After each incubation step, the cells were washed 3 times with PBS. Subsequently, the cells were incubated with primary antibody (1:200) at 4 °C for 15 h, followed by five washes with PBS. This was succeeded by incubation with the secondary antibody (1:200) at room temperature for 3 h and another 5 times washing with PBS. The cells were stained with DAPI for 10 min. Finally, the cells were observed under a laser confocal microscope, and fluorescence intensity was quantified using Image J (Excitation: 405 nm, Excitation: 488/561 nm). The primary antibodies for HIF-1α, IL-6, and TNF-α were mouse-derived, whereas those for P65 and IL-1β were rabbit-derived. Corresponding anti-mouse and anti-rabbit secondary antibodies were employed.

#### qRT-PCR analysis

Raw 264.7 cells were seeded in a 6-well plate (3 × 10^5^ cells per well) and stimulated with LPS (10 μg/mL) for 24 h. The cells were incubated with PBS, PBS + Laser, 28.8 μg/mL PDA, 40 μg/mL PDA@Pt, and 40 μg/mL PDA@Pt + Laser (1 W/cm^2^, 5 min). After 24 h of treatment, the cells from each group were collected for qRT-PCR analysis. The mRNA expression levels of IL-1β, IL-6, TNF-α, NF-κB1, NF-κB2, and Rel were detected following these steps, using GAPDH as an internal control. Total RNA was extracted with Trizol reagent, and cDNA was synthesized from RNA using the Color Reverse Transcription Kit (with gDNA Remover) as per the manufacturer's instructions. qRT-PCR was executed employing SYBR Green qPCR Master Mix on the LightCycler 96 system. The qRT-PCR conditions were as follows: initial denaturation at 95 °C for 5 min, denaturation at 95 °C for 10 s, annealing/extension at 60 °C for 30 s, for 40 cycles. Sequence-specific primer pairs are shown in Table S1. The relative mRNA expression levels were calculated using the 2 ^−ΔΔCt^ method.

#### The acute gout model and treatment in rats

All procedures involving animals were conducted in strict compliance with the Animal Management Regulations set forth by the Ministry of Health of the People’s Republic of China and adhered to the Southern Medical University’s Guidelines for the Care and Use of Experimental Animals. The male Sprague–Dawley (SD) rats used in the experiment (average weight 170 ~ 190 g) were purchased from Southern Medical University (Permit Number: 44002100036451). Prior to experimentation, these rats underwent an acclimatization period of 3–4 days. Specifically, 1.25 g of dried MSU powder was weighed and placed in a centrifuge tube, to which 36 mL 0.9% sterile sodium chloride solution and 4 mL of Tween 80 were added, resulting in a 25 mg/mL MSU suspension. The mixture was vortexed for 10 min and sonicated until it formed a suspension. Subsequently, MSU suspension (50 mg/Kg) was injected into the right ankle joint of the male SD rats. Redness, swelling, and a localized increase in temperature of the right ankle joint observed at 12 h following the injection were indicative of the successful induction of the gout model in rats. The successful modeling of SD rats were randomly divided into five groups (Saline, Saline + Laser, PDA, PDA@Pt, PDA@Pt + Laser), with five rats per group. Healthy SD rats served as the control group. For the intervention, the rats in the PDA (3.6 mg/kg) and PDA@Pt (5 mg/kg) nanomedicine groups received intra-articular injections of the respective nanomedicines into the right ankle joint. The saline and saline + Laser groups were administered the equivalent volume of saline. The saline + Laser and PDA@Pt + Laser groups were subjected to laser irradiation (808 nm, 1 W/cm^2^, 5 min) 12 and 24 h after administration. The temperature changes and infrared thermal imaging were recorded using a thermal imaging camera. The diameters of the ankle joints were meticulously measured and recorded at 12 h intervals.

#### Histological analysis

At the end of the in vivo treatment, the SD rats were humanely euthanized. We harvested tissues from the heart, liver, spleen, lung, kidney, and ankle joints for histopathological analysis. Initially, the collected tissues were thoroughly washed multiple times with saline solution. This step was followed by fixation in 4% paraformaldehyde for a duration of 24 h. Soft tissues were dehydrated after fixation with embedding clips. Rat ankle joint tissues were decalcified in 10% EDTA solution at 37 °C on a shaking incubator for 2 months. This was followed by dehydration, paraffin embedding, and sectioning into slices of 4 μm thickness. The prepared slides were baked, followed by dewaxing, rehydration, staining (employing HE, Safranin O, and immunofluorescence for HIF-1α, IL-6, IL-1β, TNF-α, NF-κB(P65)), dehydration, clearing, and mounting. Additionally, the steps for tissue immunofluorescence staining after dewaxing and hydration were the same as those in the cell immunofluorescence experiment.

#### Statistical analysis

The statistical significance of differences between different groups was analyzed using Student's t-test and one-way ANOVA (n.s. no significance, *p < 0.05, **p < 0.01, and ***p < 0.001, respectively). Data were expressed as the mean ± standard deviation (SD) (n ≥ 3).

## Results

1.1. Preparation and Characterization of PDA@Pt

**Fig. 1:**
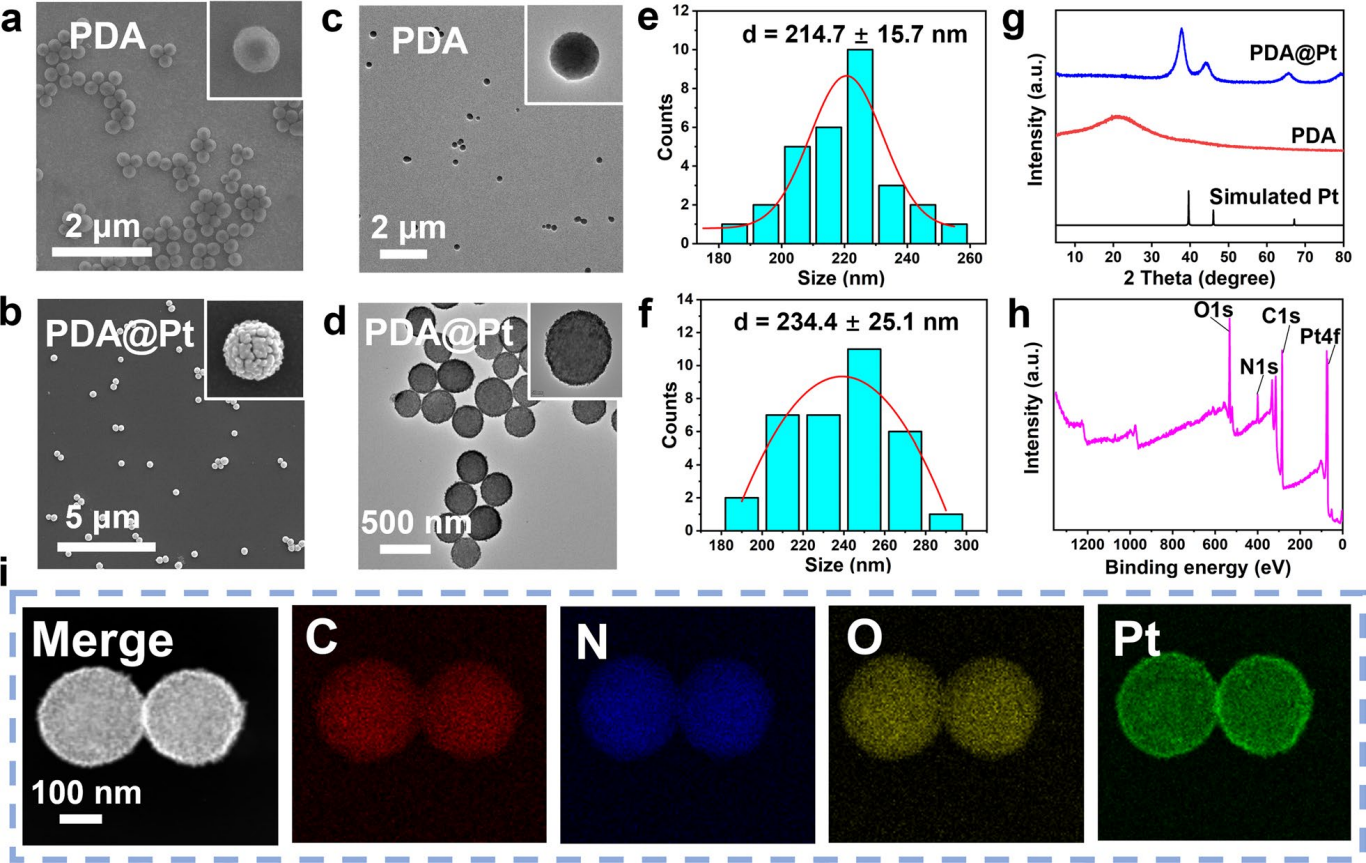
The SEM images (**a**, **b**) and TEM images (**c**, **d**) of PDA and PDA@Pt. The size distributions of (**e**) PDA and (**f**) PDA@Pt. **g** The PXRD patterns of the simulated nano Pt synthesized PDA and PDA@Pt. **h** The XPS survey spectra of PDA@Pt. **i** The elemental mapping images of PDA@Pt

The preparation of PDA@Pt nanoparticles involves two steps: Firstly, dopamine undergoes oxidative self-polymerization under alkaline conditions to form polydopamine (PDA) nanoparticles. Subsequently, the Pt^4+^ ions in K_2_PtCl_6_ are reduced using NaBH_4_ to facilitate the formation of Pt nanoparticles, which are then modified on the surface of template PDA. This process produces a core–shell nanocomposite structure of PDA@Pt. As shown in Fig. 1a, c, and e, the synthesized PDA has a uniform spherical structure with an average particle size of 214.7 ± 15.7 nm. The Pt nanoparticles are uniformly distributed on the surface of PDA. The resulting PDA@Pt has an average particle size of 234.4 ± 25.1 nm (Fig. 1b, d, and f). The average hydrodynamic diameters of PDA and PDA@Pt gradually increase, as characterized by the Dynamic Light Scattering (DLS) data (Figure S1). Furthermore, the characteristic elements of C, N, O, and Pt were detected in PDA@Pt, confirming the successful construction of PDA@Pt (Fig. 1i). The zeta potentials of PDA (− 23.2 ± 1.0 mV) and PDA@Pt (− 39.3 ± 2.8 mV) also confirmed the successful establishment of these two nanoparticles (Figure S2). There was no obvious change in the UV–Vis spectra of PDA and PDA@Pt (Figure S3). The powder X-ray diffraction (PXRD) peaks of PDA@Pt align with nano Pt particles. The diffraction peaks of nano-Pt overshadow those of PDA, indicating that a nano-Pt shell has formed on the surface of PDA (Fig. 1g). X-ray photoelectron spectroscopy (XPS) of PDA@Pt reveals that the signals from four elements, including C (1s at 285.08 eV), N (1s at 399.81 eV), O (1s at 532.09 eV) and Pt (4f at 71.28 eV, 4f7 at 74.58 eV) (Fig. 1h and Figure S4), further confirming the successful growth of the platinum shell layer [22]. Inductively coupled plasma mass spectrometry (ICP-MS) analysis shows that the mass ratio of Pt in PDA@Pt is 28.0% (Figure S5).

Stability is the key factor for applying PDA@Pt both in vitro and in vivo. Therefore, we have explored the stability of PDA and PDA@Pt in various media (water, PBS, 0.9% NaCl, and DMEM + 10% FBS). As shown in Figure S6a, both PDA and PDA@Pt exhibit remarkable dispersibility in the four different media (water, PBS, 0.9% NaCl, and 10%FBS + DMEM) in 7 h. Notably, they exhibit optimal stability in water and 10%FBS + DMEM, maintaining their integrity even after 17 h, which amply suffices for experimental purposes. In PBS and 0.9%NaCl, we observed a gradual decline in the stability of the PDA@Pt complex, accompanied by the emergence of a small number of precipitates within the timeframe of 7–17 h.

Besides, we examined the enduring stability of PDA@Pt over extended durations in various dispersive environments. Our evaluation of the colloidal dispersibility of PDA@Pt revealed that the sedimentation rate was most pronounced in a 0.9% NaCl solution, regardless of whether the conditions were maintained at room temperature or 4 °C (Figure S6b). Over a period of 7 days at ambient temperature, the sedimentation rate of PDA@Pt decreased in the following order: 0.9% NaCl, PBS, DMEM + 10% FBS, and H_2_O. However, at 4 °C, sedimentation of PDA@Pt was only observable in the 0.9% NaCl solution, whereas it remained stable in H_2_O, PBS, and DMEM + 10% FBS even after a week. As demonstrated in Figures S6c and d, the particulate size and zeta potential of PDA@Pt exhibited no substantial alterations over a period of 7 days, regardless of the temperature conditions (4 °C or room temperature) or the choice of dispersion media. The zeta potential of PDA@Pt differs across various dispersion media due to the different ion environments. However, the zeta potential of PDA@Pt within the same medium remained consistent over time.

In summary, the stability of PDA@Pt is least favorable in a 0.9% NaCl solution, while it exhibits optimal stability in H_2_O, PBS, and DMEM + 10% FBS at 4 °C, indicating that refrigeration at 4 °C is more conducive to PDA@Pt stability.

2.2. Photothermal Performance of PDA@Pt

**Fig. 2:**
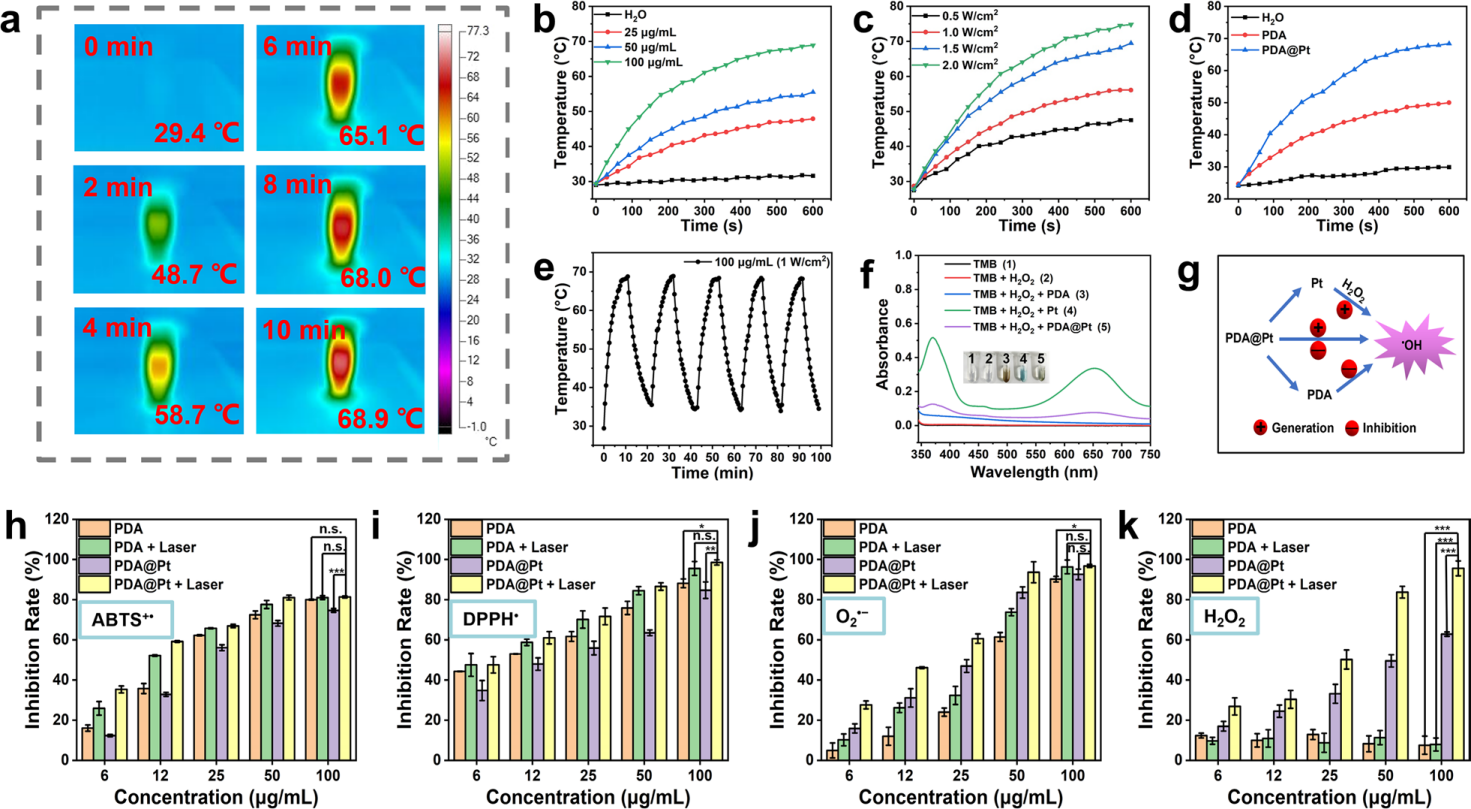
**a** The thermographic images of PDA@Pt (100 μg/mL, 808 nm, 1 W/cm^2^) at a different time (0, 2, 4, 6, 8, 10 min). **b** The photothermal effects of PDA@Pt solution with graded concentrations (0, 25, 50, 100 μg/mL). **c** The photothermal effects of PDA@Pt solution (50 μg/mL) with different power densities (0.5, 1.0, 1.5, 2.0 W/cm^2^). **d** The photothermal performance of H_2_O, PDA (72 μg/mL), and PDA@Pt (100 μg/mL). **e** The photothermal heating and cooling curves of PDA@Pt (100 μg/mL) for five cycles (808 nm, 1 W/cm^2^). **f** The ^•^OH generation by PDA (36 μg/mL), Pt (14 μg/mL) and PDA@Pt (50 μg/mL). **g** Diagram illustrating the generation and elimination of ^•^OH. The elimination of ABTS^+•^ (**h**), DPPH^•^ (**i**), O_2_^•−^ (**j**), and H_2_O_2_ (**k**) by PDA, PDA + Laser, PDA@Pt and PDA@Pt + Laser (1 W/cm^2^, 808 nm, 5 min). Data are presented as the mean ± SD (n = 3) and asterisks (*) were noted as significant differences (n.s. no significance, *p < 0.05, **p < 0.01, and ***p < 0.001)

Photothermal therapy (PTT) is a promising treatment modality for various diseases that involves the use of microwaves, radio frequencies, NIR, or visible light to activate photothermal agents. When activated, these photothermal agents can elevate their temperature as well as that of the surrounding tissues, resulting in a therapeutic effect that can inhibit abnormal tissue growth and also exhibit anti-inflammatory actions synergistically [23, 29, 30]. PDA, in particular, has a strong absorption capacity in the NIR region and high photothermal conversion efficiency [31]. As shown in Fig. 2a, b, when different concentrations of PDA@Pt (0, 25, 50, 100 μg/mL) are irradiated with NIR light (808 nm, 1 W/cm^2^) for 10 min in PBS, the temperature rise exhibits good concentration dependence which can increase to 68.9 °C at a concentration of 100 μg/mL. In addition, the temperature rise also exhibits good power dependence (0.5, 1, 1.5, 2 W/cm^2^) as shown in Fig. 2c. Under the influence of an 808 nm laser (1 W/cm^2^), when the concentration of PDA@Pt is 100 μg/mL, the temperature of the solution rapidly rises from 24.6 to 68.4 ℃ within 10 min. In contrast, the temperature of an equivalent dose of PDA increases from 24.7 to 50 ℃, indicating that the addition of Pt nanoshell enhances the photothermal capability of PDA@Pt (Fig. 2d). After five cycles of laser on/off cycling tests, the photothermal performance of the PDA@Pt did not change significantly. The photothermal conversion efficiency of PDA@Pt was found to be 32.8% (Fig. 2e and S7). These results indicate that PDA@Pt possesses excellent photothermal stability and can be effectively utilized for PTT in acute gout treatment.

3.3. ROS Scavenging Capability of PDA@Pt

During the early stage of inflammation, neutrophils engulf MSU crystals and generate ROS through the Nicotinamide Adenine Dinucleotide Phosphate (NADPH) oxidase pathway. This process suppresses the expression of antioxidants such as superoxide dismutase (SOD), resulting in oxidative stress and exacerbating inflammation [32]. We employed the ABTS antioxidant capacity assay kit to assess the overall antioxidant capability of PDA, PDA + Laser, PDA@Pt, and PDA@Pt + Laser (808 nm, 1 W/cm^2^). The green-colored product of ABTS^+•^ (UV–Vis characteristic absorption peak at 405 nm) is formed due to the oxidation of ABTS by an oxidizing agent, and antioxidants can prevent its formation. The transition from the presence to the absence of ABTS^+•^ can be observed by the changes in the UV–Vis characteristic absorption peak. The ABTS^+•^ scavenging rate of PDA was determined to be 72.4% at the concentration of 36 μg/mL. The ABTS^+•^ scavenging rate of PDA (36 μg/mL) + Laser was determined to be 77.6%. In comparison, the scavenging rate for PDA@Pt at a concentration of 50 μg/mL (which contains 36 μg/mL PDA) was 68.2%. Notably, the ABTS^+•^ scavenging rate for PDA@Pt + Laser at a concentration of 50 μg/mL was significantly increased to 81.5% (Fig. 2h). The results indicate that the capability to scavenge ABTS^+•^ primarily originates from PDA, while the photothermal heating effect significantly boosts the scavenging efficiency of PDA@Pt against ABTS^+•^.

In addition, we employed 1,1-diphenyl-2-picrylhydrazyl (DPPH^•^), which has a characteristic absorption peak at 517 nm in UV–Vis spectroscopy, to assess the capacity of PDA, PDA + Laser, PDA@Pt, and PDA@Pt + Laser (808 nm, 1 W/cm^2^) to eliminate DPPH^•^. After co-incubating the nanodrug with DPPH^•^ for 10 min, it revealed that PDA alone had a scavenging rate of 75.9% at the concentration of 36 μg/mL. The DPPH^•^ scavenging rate of PDA (36 μg/mL) + Laser was determined to be 84.5%. In comparison, PDA@Pt exhibited a reduced rate of 63.5% at the concentration of 50 μg/mL, However, when combined with laser treatment (808 nm, 1 W/cm^2^), the scavenging rate of PDA@Pt significantly increased to be 84.9% at the concentration of 50 μg/mL (Fig. 2i). Similarly, the photothermal effect significantly bolsters the capacity of PDA@Pt to scavenge DPPH^•^. Continuing our analysis, we employed the superoxide anion (O_2_^•−^) assay kit to assess the SOD activity of PDA and PDA@Pt. The results demonstrate that both PDA and Pt possess the capability to scavenge O_2_^•−^, which is synergistically enhanced when subjected to photothermal conditions (Fig. 2j).

H_2_O_2_ reacts with potassium iodide (KI) to produce I_2_/I_3_^−^, which can be quantified by measuring the characteristic absorption peak at 350 nm using UV–Vis spectroscopy [33]. Consequently, we utilized the KI method to evaluate the scavenging ability of PDA, PDA + Laser, PDA@Pt, and PDA@Pt + Laser (808 nm, 1 W/cm^2^) toward H_2_O_2_. As shown in Fig. 2k, PDA alone cannot scavenge H_2_O_2_. Thus, the degradation of H_2_O_2_ by PDA@Pt is attributed to Pt nanoparticles, and this effect is significantly amplified under photothermal conditions. It is well known that the decomposition of H_2_O_2_ by the POD activity of Pt nanoparticles can lead to the formation of ^•^OH [34]. Therefore, we employed the fluorescence method to assess the capacity of different nanosystems to scavenge ^•^OH. The generated ^•^OH can react with terephthalic acid (TA) to form a fluorescent compound TA-OH [35]. As depicted in Figure S8, the scavenging capability of PDA against ^•^OH displays concentration-dependent behavior. Moreover, our findings confirm that while Pt nanoparticles facilitate the conversion of H_2_O_2_ into ^•^OH (Fig. 2f), the PDA@Pt results showed an almost negligible amount of ^•^OH. This trend persists even at high concentrations (200 μg/mL), where only a minuscule quantity of ^•^OH is formed (Figure S9). These results suggest that PDA is capable of scavenging the majority of ^•^OH produced by Pt (Fig. 2g).

4.4. UA Degradation Activity of PDA@Pt

**Fig. 3:**
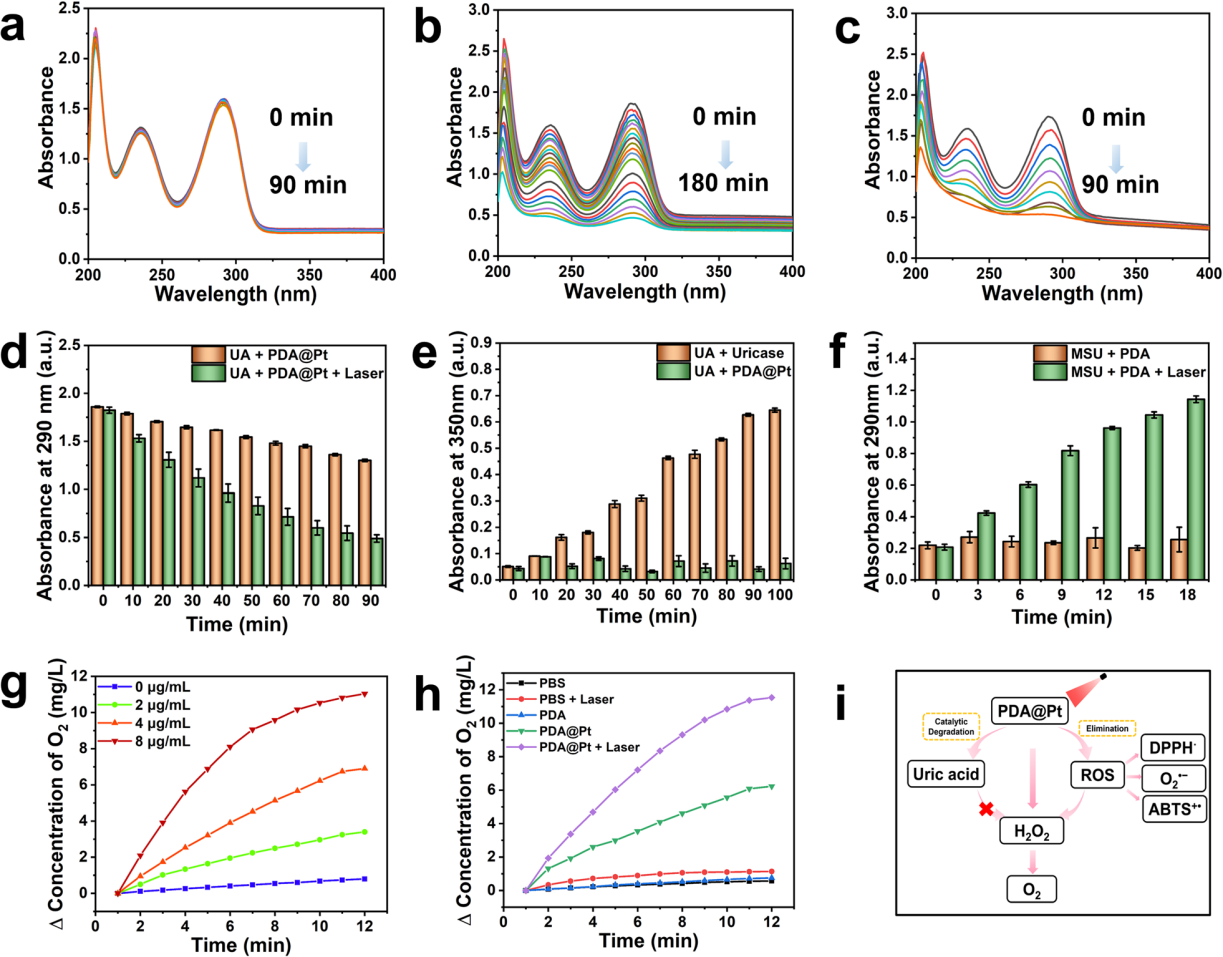
The UV–vis spectra were recorded every 10 min for PDA (**a**), PDA@Pt (**b**), and PDA@Pt + Laser (808 nm, 1 W/cm^2^) (**c**) catalyzed degradation of UA. 100 μM of UA and 15 μg/mL of corresponding NPs were used in the reaction at 37 °C. **d** The quantitative results of UA degradation by PDA@Pt or PDA@Pt + Laser (808 nm, 1 W/cm^2^). **e** The absorbance changes of I_3_^−^ produced from the reaction between H_2_O_2_ and KI, generated from the degradation of UA by PDA@Pt and uricase. (UA: 1 mM, PDA@Pt: 50 μg/mL, Uricase: 5 U/mL). **f** The absorbance changes of UA during the photothermal conversion of MSU to UA in PBS. (MSU: 0.5 mg/mL, PDA: 0.1 mg/mL, Laser: 808 nm, 1 W/cm^2^). **g** The O_2_ levels produced from the reaction of H_2_O_2_ with various concentrations of PDA@Pt. **h** The O_2_ generation curves of PBS, PBS + Laser (808 nm, 1 W/cm^2^), PDA, PDA@Pt, and PDA@Pt + Laser (808 nm, 1 W/cm^2^) for 12 min. **i** The schematic presentation of PDA@Pt + Laser (808 nm, 1 W/cm^2^) degrading UA, decomposing H_2_O_2_, and scavenging various ROS

Pt NPs serve as efficient mimics of uricase in the oxidation process of UA, catalyzing its degradation into more soluble substances without generating H_2_O_2_ [18]. It was observed that PDA alone does not exhibit UA-lowering effects (Fig. 3a and S10a), whereas the catalytic degradation of UA (UV–Vis characteristic absorption peak at 290 nm) by PDA@Pt shows time dependence (Fig. 3b). The optimal UA reduction was achieved with the mass ratio of PDA to Pt at 1:1, which is comparable to the efficacy observed at 1:0.5 (Figure S10b). Taking into account biosafety and cost-effectiveness, we opted for the PDA@Pt nanoparticles with a mass ratio of 1:0.5 for subsequent experiments. Furthermore, the degradation of UA can be significantly accelerated under the heating effect induced by the NIR laser irradiation (Fig. 3c, d). Additionally, the solubility of MSU crystals is enhanced under photothermal conditions, indirectly promoting UA degradation (Fig. 3f). It is crucial to consider that uricase generates H_2_O_2_ during the process of lowering UA levels, which could exacerbate inflammation. We utilized the KI method to investigate whether PDA@Pt generates H_2_O_2_ during the catalysis of UA degradation. As illustrated in Figure S11, uricase leads to a yellow solution due to the generation of H_2_O_2_, and its concentration increases over time (Fig. 3e). In contrast, PDA@Pt does not produce H_2_O_2_ and remains a clear and transparent solution. This suggests that PDA@Pt has the potential as an effective alternative to uricase for catalyzing the degradation of UA without the undesired generation of H_2_O_2_.

5.5. O_2_ Generation Activity of PDA@Pt

It has been reported that MSU can activate NADPH oxidase to produce H_2_O_2_ [32]. Hence, our objective was to alleviate the local hypoxic condition by degrading H_2_O_2_ in situ to generate oxygen (O_2_). We evaluated the O_2_ production ability of PDA, PDA@Pt, and PDA@Pt + Laser (808 nm, 1 W/cm^2^) through their reaction with H_2_O_2_. As shown in Fig. 3h, PDA alone did not facilitate the degradation of H_2_O_2_ to produce O_2_, whereas PDA@Pt exhibited a concentration-dependent response (Fig. 3g and S12). The yield of O_2_ was significantly augmented under the photothermal effect. For example, at the same concentration of PDA after 12 min, the generated amount of O_2_ by PBS, PDA (2.88 μg/mL), PDA@Pt (4 μg/mL, which contains 2.88 μg/mL PDA), and PDA@Pt + Laser (4 μg/mL, 808 nm, 1 W/cm^2^) were 0.55 mg/L, 0.72 mg/L, 6.08 mg/L, and 11.37 mg/L, respectively. These results indicated that PDA@Pt can catalyze the degradation of UA without producing H_2_O_2_, overcoming the limitations of uricase in degrading UA. At the same time, it decomposes H_2_O_2_ to produce O_2_, alleviating the hypoxic environment in acute gout. Furthermore, it combats the inflammatory response induced by MSU by scavenging various ROS (Fig. 3i). These results highlight the potential of PDA@Pt as an effective and safe alternative for treating gout, providing a promising strategy for the development of novel therapeutic agents against this disease.

6.6. Biocompatibility of PDA@Pt

**Fig. 4:**
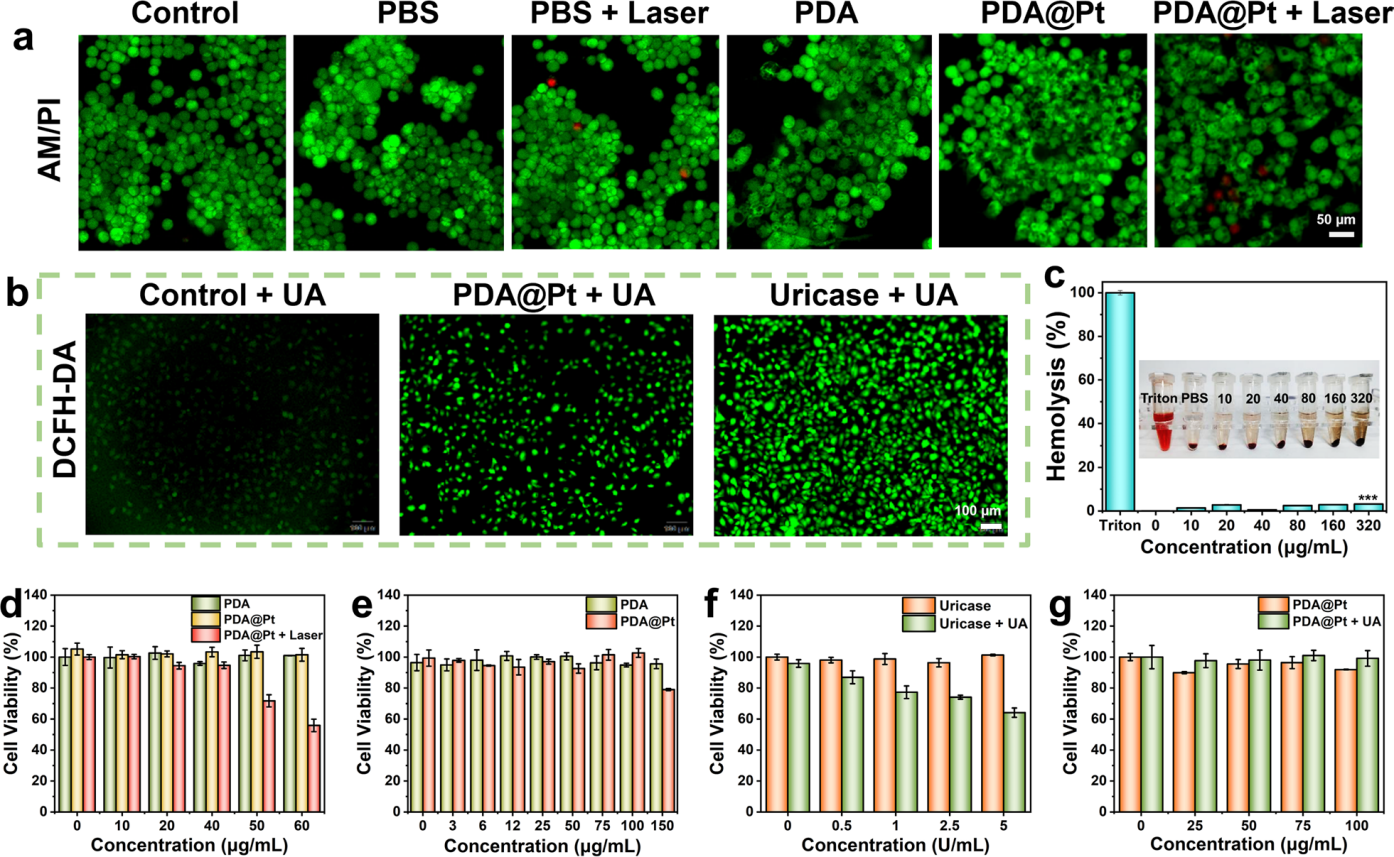
**a** The live (green)/dead (red) staining assay of LPS-induced Raw 264.7 cells after different treatments, and the scale bar is 50 μm. **b** The fluorescence images of ROS level in the HUVEC with different treatments for 24 h (Scale bar: 100 μm). **c** The hemolytic test of PDA@Pt. **d** The cell viability of LPS-induced Raw 264.7 cells treated with different concentrations (0, 10, 20, 40, 50, and 60 μg/mL) of PDA, PDA@Pt, and PDA@Pt + Laser (808 nm, 1 W/cm^2^, 5 min). **e** The cell viability of pristine Raw 264.7 cells treated with different concentrations (0, 3, 6, 12, 25, 50, 75, 100, and 150 μg/mL) of PDA and PDA@Pt. **f** The cell viability of HUVEC was treated with different concentrations (0, 0.5, 1, 2.5, and 5 U/mL) of uricase and UA (1 mM). **g** The cell viability of HUVEC was treated with different concentrations (0, 25, 50, 75, and 100 μg/mL) of PDA@Pt and UA (1 mM). (PBS: LPS; PBS + Laser: LPS + NIR)

The 3-(4,5-Dimethylthiazol-2-yl)-2,5-diphenyltetrazolium bromide (MTT) assay was employed to assess the viability of fibroblast-like synoviocytes (FLS), human umbilical vein endothelial cells (HUVEC), Raw 264.7 cells, and lipopolysaccharide (LPS)-stimulated Raw 264.7 cells following various treatments. The results revealed that the cell viability in HUVEC cells remained stable after treatment with different concentrations (0, 6, 12, 25, 50, 75, 100 μg/mL) of PDA and PDA@Pt. However, when PDA@Pt was at a concentration of 100 μg/mL, the cell viability of FLS dropped to 76.2%, indicating that PDA@Pt is essentially non-toxic to HUVEC but has mild toxicity to FLS (Figure S13a-b). Additionally, when pristine Raw264.7 cells were treated with varying concentrations (0, 3, 6, 12, 25, 50, 75, 100, and 150 μg/mL) of PDA and PDA@Pt, the PDA alone did not exhibit a significant decline in cell viability, while the PDA@Pt group showed a minor reduction at high concentrations, suggesting that the nanodrug is virtually non-toxic to normal Raw264.7 cells (Fig. 4e). Different concentrations of PDA, PDA@Pt, and PDA@Pt + Laser (808 nm, 1 W/cm^2^, 5 min) were used to treat LPS-stimulated Raw264.7 cells. As shown in Fig. 4d, the viability of the cells treated with PDA and PDA@Pt remained stable as the drug concentration increased. In contrast, the cytotoxicity in the PDA@Pt + Laser (808 nm, 1 W/cm^2^) group was decreased, with cell viability reaching 94.7% at a concentration of 40 μg/mL and dropping below 80% at 50 μg/mL (Fig. 4d). Therefore, a concentration of 40 μg/mL of PDA@Pt was employed in the subsequent experiments. To visually observe the live and dead cells induced by different nanocomposites, we stained the cells with Calcein AM (acetoxymethyl ester of calcein, green fluorescence indicates live cells) and propidium iodide (PI, red fluorescence indicates dead cells) [36]. As shown in Fig. 4a, it is evident that there are almost no dead cells in the control, PBS, PBS + Laser, PDA, and PDA@Pt groups, while only a few dead cells are observed in the PDA@Pt + Laser group. This observation aligns with the results of the MTT assay, suggesting that our treatment does not directly kill inflammatory cells but rather modulates their state to rescue cell damage. We further evaluate the hemolysis rate of PDA@Pt to explore its biocompatibility and safety. Even at a high concentration of 320 μg/mL, the hemolysis rate of PDA@Pt was only 3.2%, demonstrating the excellent safety profile of the nanodrug (Fig. 4c).

Furthermore, the HUVEC cells were incubated with different concentrations of uricase (0, 0.5, 1, 2.5, 5 U/mL), PDA@Pt (0, 25, 50, 75, 100 μg/mL), and UA (1 mM) for 24 h. The results showed that the concentration of uricase reached 5 U/mL, and the cell viability decreased to only 64.1%, indicating that the H_2_O_2_ generated through the degradation of UA by uricase could effectively induce cell death (Fig. 4f, g). In contrast, when the concentration of PDA@Pt reached 100 μg/mL, the cell viability was as high as 99.2%, indicating that PDA@Pt had no significant impact on cell viability. ROS probes further confirmed that uricase generated a higher level of ROS during UA degradation, whereas PDA@Pt does not (Fig. 4b). Overall, PDA@Pt is a nanomedicine with excellent biocompatibility and minimal toxicity.

7.7. The capability of PDA@Pt in generating intracellular oxygen and eliminating intracellular ROS

**Fig. 5:**
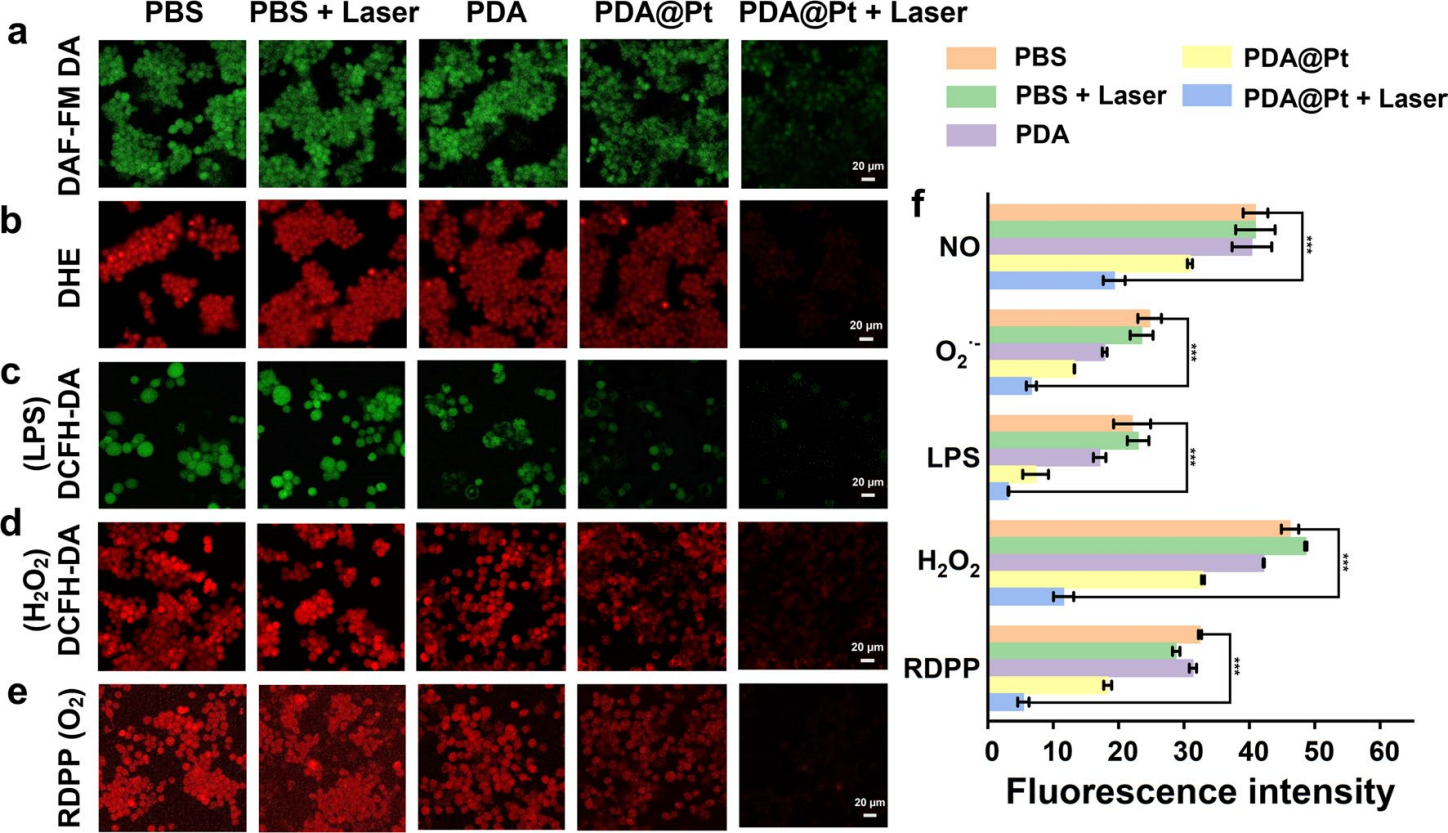
The fluorescence images of NO level (**a**), O_2_^•−^ level (**b**), ROS level induced by LPS (c), ROS level induced by H_2_O_2_ (**d**), and O_2_ level (**e**) in the LPS-induced Raw 264.7 cells with different treatments for 24 h (Scale bar: 20 μm). **f** The quantitative analysis of intracellular fluorescence intensity is based on (**a**–**e**). Data are presented as the mean ± SD (n = 3) and asterisks (*) were noted as significant differences (n.s. no significance, *p < 0.05, **p < 0.01, and ***p < 0.001). (PBS: LPS; PBS + Laser: LPS + NIR)

Research has demonstrated that persistently elevated levels of UA and the accumulation of MSU crystals can disrupt the balance between oxidative processes and antioxidant defense systems within the body, ultimately leading to oxidative stress. This imbalance can trigger the production of an array of ROS, such as NO, O_2_^•−^, H_2_O_2_, etc., which play important roles in the pathophysiological processes that drive gout inflammation [6]. To assess the capacity of PDA@Pt to scavenge these ROS, we employed 3-amino,4-aminomethyl-2', 7'-fluorescein, diacetate (DAF-FM DA) as a fluorescent probe to monitor the level of nitric oxide (NO) within cells. DAF-FM DA is distinguished by its specificity for NO and its ability to permeate live cell membranes. Inside the cell, it is enzymatically cleaved by cytoplasmic esterases to form DAF-FM, which remains intracellular since it cannot cross the membrane. Under normal conditions, DAF-FM exhibits minimal fluorescence. However, its interaction with NO leads to the formation of a fluorescent benzotriazole derivative that emits intense green fluorescence [37]. As shown in Fig. 5a, we observed that the groups of PBS, PBS + Laser (808 nm, 1 W/cm^2^), and PDA exhibited pronounced green fluorescence, indicating high intracellular NO levels. However, following treatment with the PDA@Pt, there was a noticeable reduction in fluorescence emission, which was further diminished when combined with laser exposure (808 nm, 1 W/cm^2^). These aforementioned findings also imply that the NO scavenging effect is primarily attributed to the Pt nanoparticles present in PDA@Pt.

Dihydroethidium (DHE) is a commonly used fluorescent probe for detecting O_2_^•−^. This dye readily penetrates cells and undergoes dehydrogenation to form ethidium bromide in the presence of intracellular O_2_^•−^. Ethidium bromide then binds to RNA or DNA to result in red fluorescence. The intensity of the fluorescence is directly proportional to the level of O_2_^•−^ within the cell [38]. As illustrated in Fig. 5b, the red fluorescence indicative of O_2_^•−^ levels in Raw 264.7 cells was particularly strong when treated with PBS and PBS + laser (808 nm, 1 W/cm^2^). However, following treatment with PDA and PDA@Pt, there was a gradual reduction in the intensity of the red fluorescence, with virtually no fluorescence observed in the group treated with PDA@Pt combined with laser exposure (808 nm, 1 W/cm^2^).

Subsequently, we employed 2',7'-dichlorodihydrofluorescein diacetate (DCFH-DA) as a fluorescent probe to evaluate the intracellular levels of ROS. DCFH-DA is internalized by the cell and then deacetylated by cellular esterases to generate 2',7'-dichlorodihydrofluorescein (DCFH), which is then oxidized by ROS to yield the green fluorescent compound 2',7'-dichlorofluorescein (DCF) [39]. As illustrated in Fig. 5c, the green fluorescence which signifies the levels of ROS, was particularly prominent in Raw264.7 cells treated with LPS in PBS and PBS + laser (808 nm, 1 W/cm^2^). However, following treatment with PDA and PDA@Pt, there was a noticeable decrease in the fluorescence intensity, highlighting their effective capacity to clear ROS. The fluorescence was almost completely absent in the group of PDA@Pt + laser (808 nm, 1 W/cm^2^). Additionally, we investigated the impact of PDA@Pt on the ROS generation in Raw264.7 cells induced by H_2_O_2_. After stimulation with H_2_O_2_, a significant increase in green fluorescence was observed in the PBS, indicating severe oxidative stress and elevated ROS levels in the stimulated Raw 264.7 cells. In contrast, there was minimal change in the group treated with laser alone (808 nm, 1 W/cm^2^) and PDA, whereas ROS levels were reduced in the PDA@Pt groups. Under the combined effect of PDA@Pt and laser (808 nm, 1 W/cm^2^), the green fluorescence was nearly undetectable (Fig. 5d).

[Ru(dpp)_3_] Cl_2_ (denoted as RDPP) is an oxygen-sensitive compound that emits strong fluorescence under hypoxic conditions. Nevertheless, its fluorescence tends to be partially quenched in the presence of high levels of O_2_ [40]. In Raw 264.7 cells stimulated with LPS and subjected to hypoxic conditions, the intensity of the red fluorescence is pronounced, indicating a state of severe hypoxia. Upon treatment with PDA@Pt, the red fluorescence gradually weakened. However, when PDA@Pt is combined with laser therapy (808 nm, 1 W/cm^2^), the red fluorescence nearly disappeared. This indicates that laser irradiation can enhance the capacity of Pt to produce O_2_, effectively ameliorating the hypoxic state of the cells (Fig. 5e).

In conclusion, PDA@Pt, as a nanozyme combined with photothermal therapy, demonstrates exceptional ROS scavenging and O_2_ generation capabilities, which is advantageous for the in vivo management of acute gout.

8.8. The ability of PDA@Pt to promote mitochondrial homeostasis

**Fig. 6:**
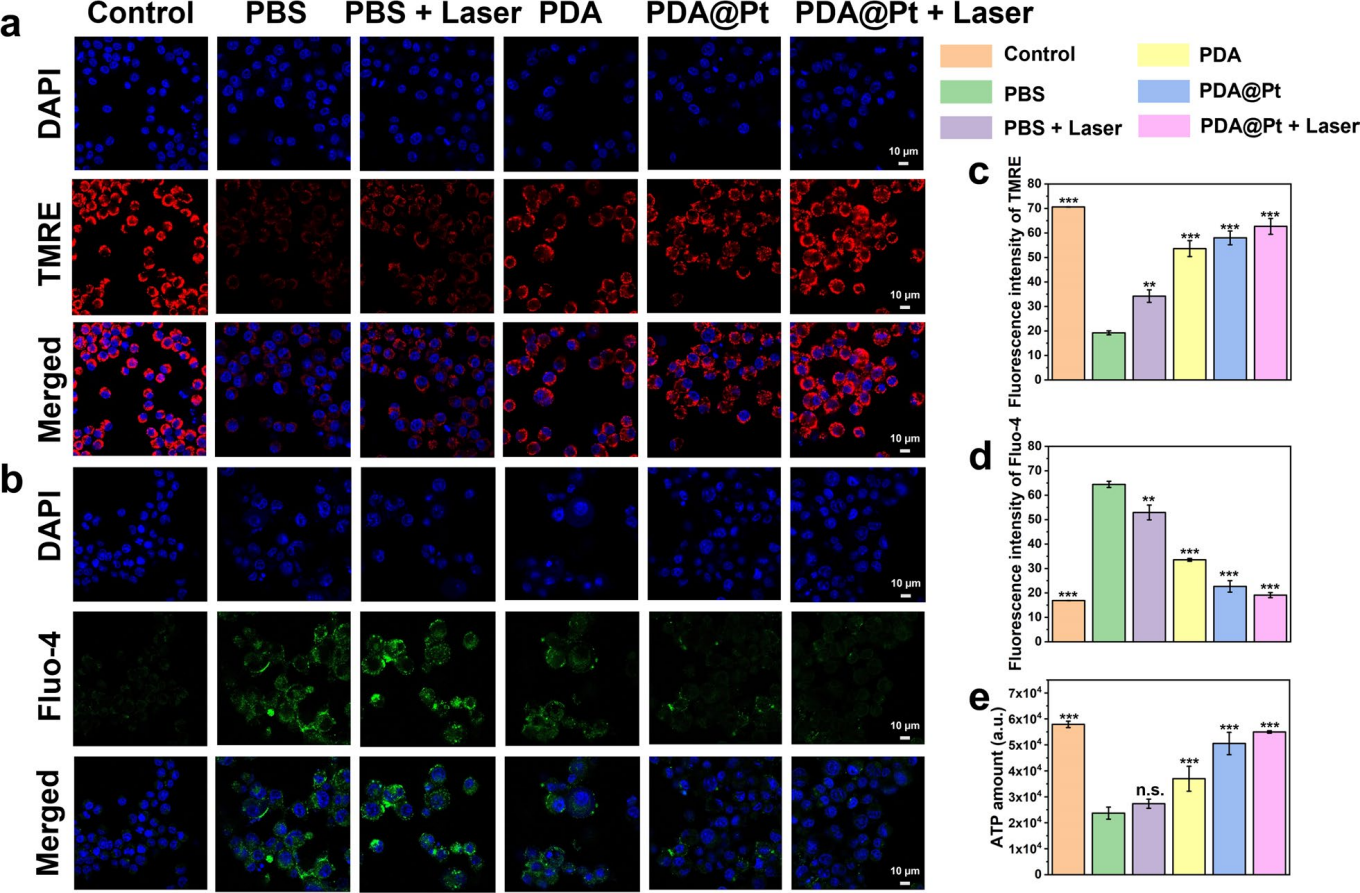
The immunofluorescent imaging of TMRE (**a**) and Fluo-4 (**b**) in LPS-induced Raw 264.7 cells after different treatments (Scale bars: 10 μm). The quantitative analysis of fluorescence intensity of TMRE (**c**) and Fluo-4 (**d**) in LPS-induced Raw 264.7 cells after different treatments. The ATP levels (**e**) in LPS-induced Raw 264.7 cells with different treatments. Data are presented as the mean ± SD (n = 3) and asterisks (*) were noted as significant differences (n.s. no significance, *p < 0.05, **p < 0.01, and ***p < 0.001). (PBS: LPS; PBS + Laser: LPS + NIR)

Mitochondrial homeostasis is susceptible to disturbances from various factors, including inflammation, infection, and hypoxia, which can lead to mitochondrial dysfunction. This dysfunction manifests as a decline in membrane potential, an elevated calcium influx, and a decrease in ATP synthesis [41, 42]. Research has indicated that MSU can initiate immune inflammation and cause mitochondrial damage in macrophages [25]. Besides, oxidative stress reactions can also damage mitochondria [43]. To elucidate the effect of PDA@Pt on restoring mitochondrial membrane potential, tetramethylrhodamine, ethyl ester (TMRE), a cell-permeable orange-red cationic fluorescent probe, was employed for monitoring mitochondrial membrane potential alterations in cells. Under normal physiological conditions, mitochondria exhibit a substantial negative charge, which causes the accumulation of TMRE within the mitochondrial matrix and produces intense orange-red fluorescence. However, during apoptosis, the mitochondrial membrane potential is lost and the mitochondrial permeability transition pore (MPTP) remains open, allowing TMRE to escape into the cytoplasm, resulting in a significant reduction of orange-red fluorescence within the mitochondria [44]. As demonstrated in Fig. 6a, c, the control displays intense red fluorescence, indicating a normal mitochondrial membrane potential. In contrast, LPS-induced Raw 264.7 cells (PBS) display significantly reduced fluorescence, suggesting that LPS has impaired the mitochondrial membrane potential of Raw 264.7 cells. Post-treatment with different modalities (PBS + laser (808 nm, 1 W/cm^2^), PDA, PDA@Pt, PDA@Pt + laser (808 nm, 1 W/cm^2^)), a progressive increase in red fluorescence intensity is observed. These observations conclusively demonstrate that PDA@Pt can restore the mitochondrial membrane potential compromised by LPS, and the NIR light alone also has a certain regulatory effect.

Fluo-4 acetoxymethyl ester (Fluo-4 AM) is a cell-permeable fluorescent calcium indicator. It undergoes hydrolysis by intracellular esterase, releasing Fluo-4, which binds to calcium with a K_d_ value of 345 nM and emits green fluorescence [45]. LPS-induced Raw264.7 cells (PBS) show an increase in intracellular calcium influx. Nevertheless, following various treatments [PDA, PDA@Pt, PDA@Pt + laser (808 nm, 1 W/cm^2^)], the calcium influx decreases, indicating an improvement in calcium homeostasis (Fig. 6b, d). This effect is particularly pronounced under photothermal conditions, indicating that both PDA and PDA@Pt can effectively regulate calcium homeostasis, with further enhancement under photothermal stimulation. Additionally, we measured the intracellular ATP levels. LPS-induced Raw264.7 cells (PBS) exhibited a significant decrease in intracellular ATP. In contrast, co-incubation with PBS + laser (808 nm, 1 W/cm^2^), PDA, PDA@Pt, and PDA@Pt + laser (808 nm, 1 W/cm^2^) led to a gradual increase in ATP levels (Fig. 6e). Notably, the PDA@Pt + laser (808 nm, 1 W/cm^2^) treatment substantially alleviated the reduction in ATP levels in LPS-induced Raw264.7 cells.

The mitochondrial membrane is rich in antioxidant enzymes on both sides, such as SOD and glutathione peroxidase (GPX). The disruption of mitochondrial membrane structure and function can attenuate the activity of these enzymes, resulting in oxidative stress-induced damage to the mitochondria [46]. However, PDA@Pt demonstrates a remarkable capability in restoring mitochondrial membrane potential, reducing calcium influx, and enhancing intracellular ATP synthesis. This protective mechanism is crucial for maintaining mitochondrial function and safeguarding mitochondria against oxidative stress.

9.9. The HIF-1α-regulating and anti-inflammatory capability of PDA@Pt

**Fig. 7:**
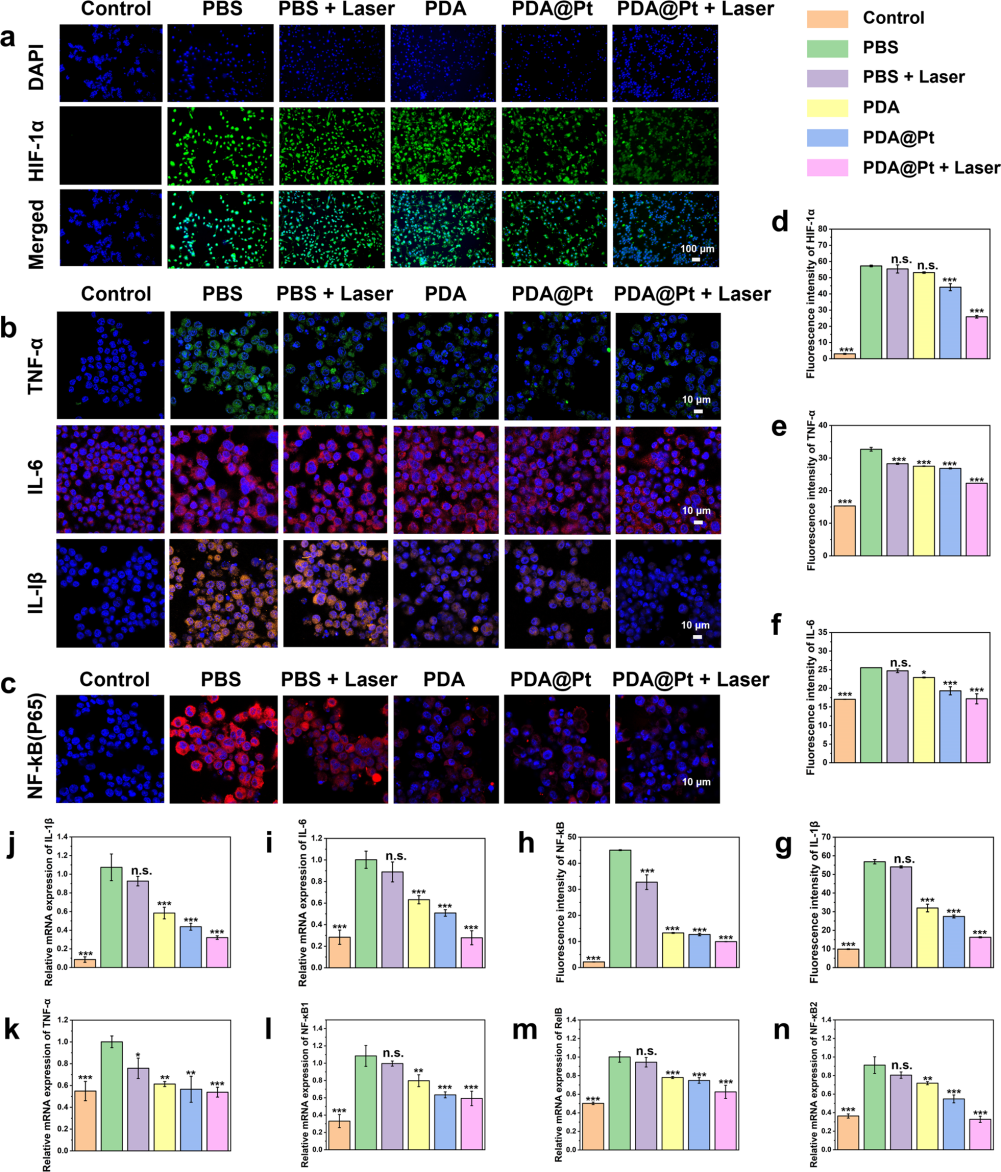
The fluorescence images of HIF-1α level (**a**), TNF-α (green), IL-6 (red) and IL-1β (orange) (**b**), NF-κB (P65) level (**c**) in the LPS-induced Raw 264.7 cells with different treatments for 24 h (Scale bar: 100 μm). The quantitative analysis of fluorescence intensity of HIF-1α (**d**), TNF-α (**e**), IL-6 (**f**), IL-1β (**g**) and NF-κB (**h**). The relative mRNA expression of TNF-α (**k**), IL-6 (**i**), IL-1β (**j**), NF-κB1 (**l**), RelB (**m**), and NF-κB2 (**n**) in the LPS-induced Raw 264.7 cells under various treatments and were quantified by qRT-PCR. Data are presented as the mean ± SD (n = 3) and asterisks (*) were noted as significant differences (n.s. no significance, *p < 0.05, **p < 0.01, and ***p < 0.001). (PBS: LPS; PBS + Laser: LPS + NIR)

Hypoxia-inducible factor-1 (HIF-1) is a heterodimeric transcription factor composed of α and β subunits that regulate cellular adaptive responses to hypoxia [47]. The intracellular O_2_ levels inversely affect the abundance of HIF-1α: its concentration rises under hypoxic conditions and falls in elevated O_2_ levels [48]. In LPS-stimulated mouse macrophages, HIF-1α enhances the production of pro-inflammatory cytokines through NF-κB mediated pathways. Direct pathogen contact in mouse macrophages leads to the upregulation of HIF-1α by the activation of the NF-κB pathway. This activation, in turn, stimulates the transcription of HIF-1α, thus forming a regulatory feedback loop [49]. Moreover, HIF-1α plays a crucial role in promoting the activation of classical M1 macrophages and regulating phosphofructokinase activity, thereby impacting the expression of inflammation-related genes [50]. In the case of acute gout, which is characterized by simultaneous hypoxia and inflammation, the modulation of HIF-1α expression becomes crucial for therapeutic intervention [51, 52]. Our studies with LPS-induced Raw 264.7 cells demonstrated significant green fluorescence, indicating heightened HIF-1α expression and severe intracellular hypoxia (Fig. 7a, d). Treatment with either NIR irradiation alone or PDA alone proved insufficient in mitigating this hypoxic state. However, the diminished green fluorescence in PDA@Pt treated cells suggests an obvious increase in O_2_ levels. This effect is further amplified by the combination of laser irradiation (808 nm, 1 W/cm^2^), signifying enhanced O_2_ production and a consequent reduction in HIF-1α expression. These findings highlight the potential of PDA@Pt as a therapeutic agent, particularly in its ability to downregulate HIF-1α and significantly ameliorate local hypoxic conditions. Furthermore, this reduction in HIF-1α is important in suppressing the NF-κB signaling pathway, thereby augmenting its anti-inflammatory effects.

NF-κB, an essential target in the treatment of inflammatory diseases, plays a vital role in biological processes including immunity and apoptosis. The genes RelB, NF-κB1, and NF-κB2 genes are key components of the NF-κB family. During acute gout episodes, MSU, similar to danger signals, are recognized by specific receptors, triggering the activation of the NF-κB signaling pathway. This activation is crucial as it escalates the release of downstream pro-inflammatory cytokines such as IL-1β, TNF-α, and IL-6 [53]. Understanding this pathway is key to developing novel therapeutic strategies targeting inflammation and its underlying mechanisms. In our research, the effectiveness of PDA@Pt as an anti-inflammatory agent was confirmed through comprehensive real-time fluorescence quantitative analysis (qRT-PCR) and immunofluorescence experiments. These tests assessed the gene expression levels associated with inflammation. As depicted in Fig. 7i-n, LPS-induced Raw 264.7 cells (PBS) displayed marked inflammatory characteristics compared to the control, including a notable increase in pro-inflammatory cytokines IL-1β, IL-6, and TNF-α, as well as abnormal activation of the NF-κB signaling pathway. However, various treatments, including PBS + laser (808 nm, 1 W/cm^2^), PDA, PDA@Pt, and PDA@Pt + laser (808 nm, 1 W/cm^2^), resulted in a marked reduction in the expression levels of these pro-inflammatory cytokines. Simultaneously, the expression levels of genes involved in the NF-κB signaling pathway (ΚB1, ΚB2, RelB) appeared to be normalized. Notably, the PBS + laser group exhibited a decrease in pro-inflammatory factors compared to the PBS, suggesting that NIR light alone has a certain anti-inflammation effect. Furthermore, immunofluorescence staining distinctly demonstrated a gradual decrease in pro-inflammatory biomarkers following diverse treatments (Fig. 7b and e–g). At the same time, NF-κB activation assays were conducted utilizing RelA (p65) protein expression as a biomarker to determine the activation status of the NF-κB signaling pathway [54]. As shown in Fig. 7c and h, LPS can upregulate the expression of P65 protein in Raw264.7 cells, thereby activating the NF-κB pathway. In contrast, subsequent treatments (PBS + laser (808 nm, 1 W/cm^2^), PDA, PDA@Pt, PDA@Pt + laser (808 nm, 1 W/cm^2^)) led to a downregulation of P65 expression and a gradual inhibition of the NF-κB signaling pathway. These findings demonstrate that the various components of the nanosystem can effectively inhibit NF-κB signaling, reduce the expression of inflammatory factors, and highlight the significant enhancement of anti-inflammatory effects through NIR irradiation. Consequently, PDA@Pt as a potent modulator of inflammatory responses, presenting novel avenues for anti-inflammatory treatments in nanomedicine.

10.10. Therapeutic effect of PDA@Pt in vivo

**Fig. 8:**
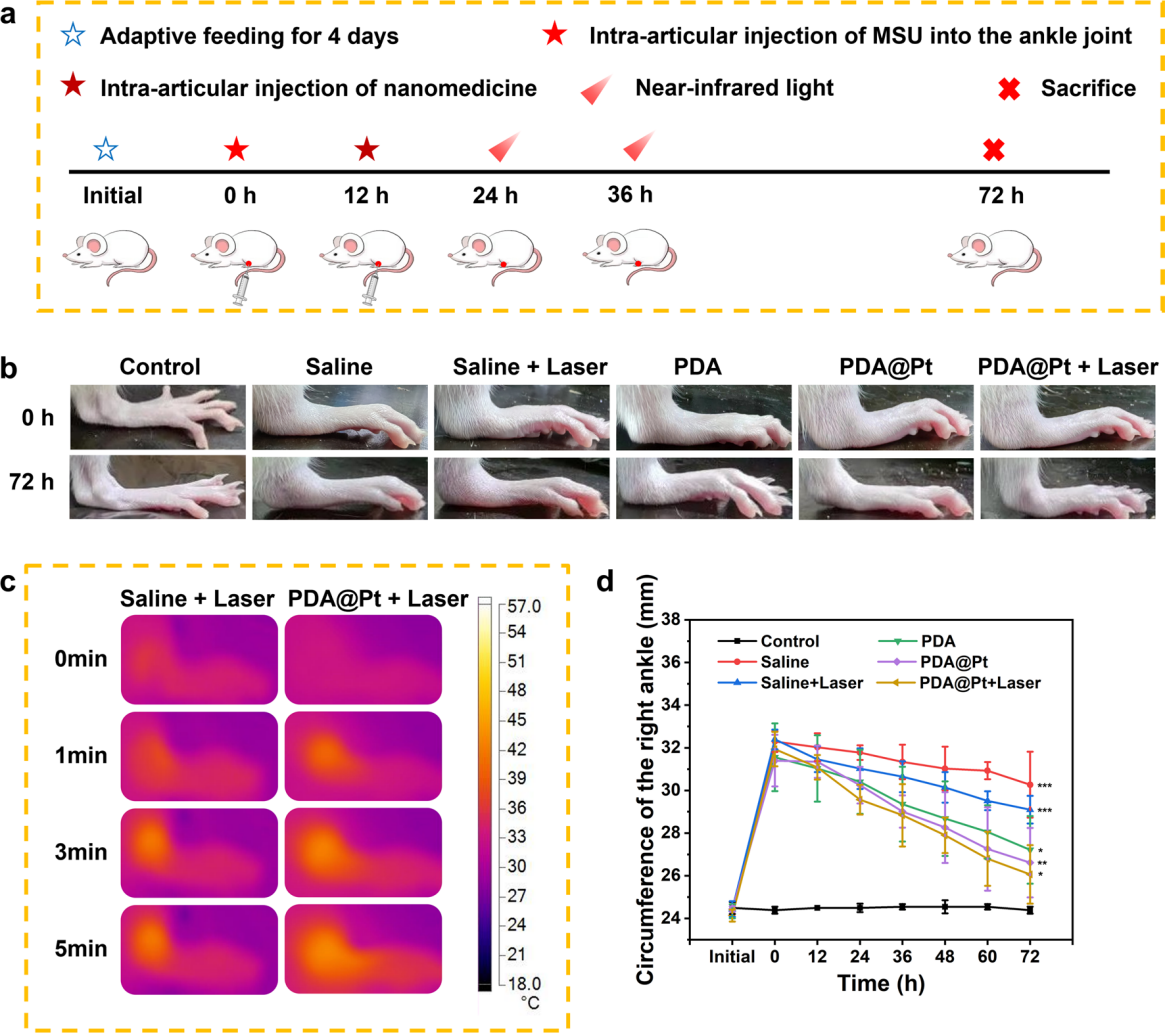
**a** Schematic representation of the treatment protocol. **b** The representative photographs of the ankle joints of SD rats after different treatments. **c** The thermal images of the ankle joints of acute gout rats treated with saline + Laser and PDA@Pt + Laser, respectively. **d** The changes in the circumference of the right ankle joint of SD rats with different treatments. Data are presented as the mean ± SD (n = 5) and asterisks (*) were noted as significant differences (n.s. no significance, *p < 0.05, **p < 0.01, and ***p < 0.001)

To study the in vivo efficacy of PDA@Pt in the treatment of acute gout in rats, MSU was injected into the ankle joints of SD rats on the first day. After injection of 12 h, the rats exhibited pathological symptoms in their ankle joints, characterized by redness, swelling, heat, and pain. According to our treatment protocol, normal rats (n = 5) were designated as the blank group. The successful establishment of the acute gout model in SD rats (n = 25) was randomly assigned to five different treatment groups: saline, saline + laser, PDA, PDA@Pt, and PDA@Pt + laser. Each group received its designated treatment as described in our experimental design (Fig. 8a). Moreover, the saline + Laser and PDA@Pt + Laser groups underwent intra-articular injection of saline and PDA@Pt, respectively, into the joints of acute gout rats. Subsequently, the rats were exposed to NIR light (808 nm, 1 W/cm^2^) for PTT, and the temperatures, along with thermal images of the ankle joints, were captured at different time intervals using a thermal imaging camera. As shown in Fig. 8c, the temperature of the ankle joints gradually increased with the duration of light exposure, and the temperature elevation in the PDA@Pt + Laser group was notably more pronounced compared to the saline + laser group. Within 5 min, the maximum temperature in the PDA@Pt + Laser group reached 43 ℃, while the highest temperature in the saline + Laser group was 39.3 ℃. Results demonstrated that there were no substantial changes observed in the control group, whereas the saline group continued to exhibit severe swelling after 72 h treatment. By contrast, the saline + Laser and PDA treatment groups showed a certain degree of reduction in swelling. In the PDA@Pt group, the condition of the ankle joint nearly normalized, although bony markers were not distinctly visible. Remarkably, the ankle joint of the PDA@Pt + Laser group had fully reverted to a normal condition, displaying clear and well-defined bony landmarks (Fig. 8b). we monitored the local temperature changes in the joints of acute gout SD rats before and after treatment (Figure S14). Post-treatment, a gradual decrease in joint temperature was observed across all groups, with the most substantial reduction noted in the PDA@Pt + Laser group. While there was an improvement in the temperature within the model group, the reduction in the nanoparticle-treated groups was more pronounced, underscoring the ability of PDA@Pt to alleviate the pathological characteristic of increased joint temperature in acute gout rats. Furthermore, we recorded changes in ankle joint swelling for each group over the 72 h treatment period, clearly demonstrating that PDA@Pt combined with laser irradiation had the best therapeutic effect (Fig. 8d). These findings indicate that PDA@Pt possesses a distinct therapeutic effect on the MSU crystal-induced acute gout rat model.

11.11. The Anti-inflammatory Effects and Safety of PDA@Pt in Vivo

**Fig. 9:**
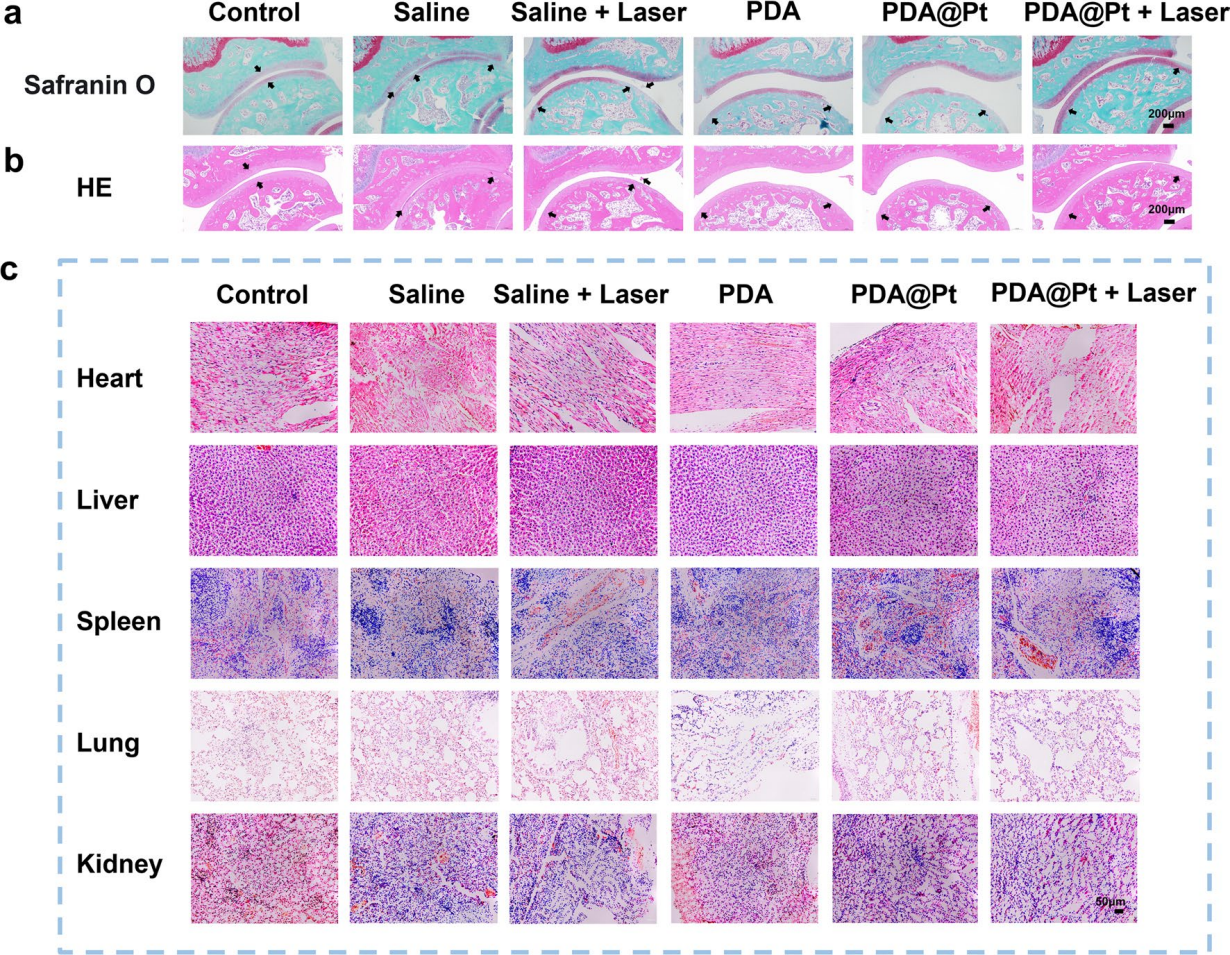
**a** Safranin-O and (**b**) H&E staining of cartilage from acute gout ankle joints (Scale bar: 200 μm). **c** The H&E staining images of the heart, liver, spleen, lung, and kidney from the acute gouty rats with different treatments (Scale bar: 50 μm)

The accumulation of MSU in joints and adjacent tissues activates tissue-resident macrophages, which phagocytose MSU, leading to the release of ROS and inflammatory mediators such as IL-1β and IL-6 [55]. Consequently, we undertook a thorough assessment of the therapeutic impact of various treatments on acute gout to gauge the efficacy of nanomedicine. Inflammatory conditions in the ankle joints markedly decreased in the groups of PDA, PDA@Pt, and PDA@Pt + Laser as assessed by Hematoxylin and Eosin (H&E) staining (Fig. 9b). Additionally, Safranin-O staining of the ankle joints revealed cartilage tissue destruction and severe synovial proliferation in the saline group. The PDA@Pt demonstrated improvements in synovial proliferation and cartilage destruction, while the joint space and cartilage morphology returned to normal in the PDA@Pt + Laser (Fig. 9a). To explore the regulatory effects and mechanism of PDA@Pt on joint inflammation, we performed immunofluorescence assays on the ankle joints, assessing changes in inflammatory markers (IL-6, IL-1β, TNF-α) and the inflammatory signaling pathway (NF-κB(P65)). Results indicated that, compared to the saline group, treatment with PDA@Pt + Laser reduced the expression of inflammatory factors (IL-6, IL-1β, TNF-α), HIF-1α and P65. This suggests that PDA@Pt + Laser exerts excellent therapeutic efficacy on acute gout in rats by inhibiting the NF-κB signaling pathway and downregulating inflammatory factor expression (Figure S15). Lastly, H&E staining was performed on the major organs (heart, liver, spleen, lungs, kidneys) of rats in different treatment groups (Fig. 9c). As illustrated, the results indicate the favorable in vivo safety of the nanomedicines in all groups.

## Discussion

Gout is a prevalent disease resulting from the accumulation of MSU crystals in joints or soft tissues, typically presenting as rapid-onset acute monoarthritis with symptoms such as intense joint pain, erythema, increased local temperature, swelling, and functional impairment [56]. The condition is often attributed to excessive crystallization due to hyperuricemia, provoking an innate immune response by irritating the synovial membrane and adjacent tissues. While some patients exhibit classic symptoms, their serum urate levels may not be significantly elevated or could even be within the normal range [57–59]. Acute gouty arthritis treatment focuses on symptom relief, anti-inflammatory measures, and analgesia, with urate-lowering therapy initiated 2 to 4 weeks post-attack resolution [60]. In this research, PDA@Pt demonstrated effectiveness in treating MSU-induced acute gout through its photothermal properties, synergistic UA reduction, ROS scavenging, and anti-inflammatory actions. Although PDA@Pt has demonstrated the ability to catalyze UA degradation in vitro, its effectiveness in vivo in MSU-induced acute gout rat models remains uncertain. This is because the model is created by directly injecting MSU into the joint cavity, which does not mimic the natural induction of gout by UA saturation in the body, making it challenging to conclusively determine if PDA@Pt can treat acute gout by breaking down UA. PDA@Pt may be more suited for acute gout cases with normal or slightly elevated serum UA levels.

## Conclusion

In conclusion, we have successfully engineered a nanomedicine of PDA@Pt, which leverages mild photothermal enhancement to synergistically improve the pathological characteristics of acute gout by reducing UA and anti-inflammatory action. Under the photothermal influence, PDA@Pt significantly degrades UA, ameliorates hypoxia and promotes mitochondrial repair. Concurrently, its dual action in scavenging ROS and inhibiting inflammatory signaling pathways leads to the suppression of pro-inflammatory cytokine expression. Importantly, the excellent biocompatibility and minimal toxicity of PDA@Pt render it a highly promising uric-acid-lowering and anti-inflammatory agent for the treatment of acute gout.

### Supplementary Information

Supplementary Material 1.

## Data Availability

All data generated or analyzed during this study are included in this published article [and its supplementary information files].

## Abbreviations

MSU: Monosodium urate
UA: Uric acid
ROS: Reactive oxygen species
NSAIDs: Non-steroidal anti-inflammatory drugs
H_2_O_2_: Hydrogen peroxide
Pt: Platinum
Pd: Palladium
POD: Peroxidase
CAT: Catalase
SOD: Superoxide dismutase
MOFs: Metal–organic frameworks
SiO_2_: Silicon dioxide
PDA: Polydopamine
PDA@Pt: Polydopamine-platinum
K_2_PtCl_6_: Potassium chloroplatinate
NaBH_4_: Sodium borohydride
LPS: Lipopolysaccharides
DAPI: 4′6-Diamidino-2-phenylindole
MTT: 3-(4,5-Dimethylthiazol-2-yl)-2,5-diphenyltetrazolium bromide
KI: Potassium iodide
UV–Vis: Ultraviolet–visible
PXRD: Powder X-ray diffraction
XPS: X-ray photoelectron spectroscopy
TEM: Transmission electron microscope
PTT: Photothermal therapy
NADPH: Nicotinamide Adenine Dinucleotide Phosphate
FLS: Fibroblast-like synoviocytes
HUVEC: Human umbilical vein endothelial cells
DAF-FM DA: 3-Amino,4-aminomethyl-2', 7'-fluorescein, diacetate
DHE: Dihydroethidium
DCFH-DA: 2',7'-Dichlorodihydrofluorescein diacetate
RDPP: [Ru(dpp)_3_] Cl_2_
TMRE: Tetramethylrhodamine, ethyl ester
GPX: Glutathione peroxidase
HIF-1: Hypoxia-inducible factor-1
