# Supplementary material for: Mild hyperthermia enhanced synergistic uric acid degradation and multiple ROS elimination for an effective acute gout therapy
Source: J Nanobiotechnology. 2024 May 22;22:275. doi: 10.1186/s12951-024-02539-9 (PMC11112921; doi:10.1186/s12951-024-02539-9)
Supplement: Supplementary file 1 — Supplementary Material 1. [file 12951_2024_2539_MOESM1_ESM.docx]

**Mild Hyperthermia Enhanced Synergistic Uric Acid Degradation and Multiple ROS Elimination for an Effective Management of Acute Gout**

Pei Zhao^1, a^, Hua-Zhong Hu^1, a^, Xiao-Tong Chen^2^, Qi-Yun Jiang^1^, Xue-Zhao Yu^1^, Xiao-Lin Cen^1^, Shi-Qing Lin^1^, Sui-qing Mai^1^, Wei-lin Pang^3^, Jin-Xiang Chen^2,^ * and Qun Zhang^1,^ *

^1^P. Zhao, H.Z. Hu, Q.Y. Jiang, X.Z. Yu, X.L. Cen, S.Q. Lin, S.Q. Mai, Q. Zhang

Guangdong Provincial Key Laboratory of Bone and Joint Degeneration Diseases

The Third Affiliated Hospital, Southern Medical University

Office of Clinical Trial of Drug

Guangzhou 510663, Guangdong, China

E-mail: zq1979@smu.edu.cn

^2^X.T. Chen, J.X. Chen

NMPA Key Laboratory for Research and Evaluation of Drug Metabolism

Guangdong Provincial Key Laboratory of New Drug Screening

School of Pharmaceutical Sciences

Southern Medical University

Guangzhou 510515, Guangdong, China

E-mail: jxchen@smu.edu.cn

^3^W.L. Pang

School of Chinese Medicine

Southern Medical University

Guangzhou 510515, China

^a^ These authors contributed equally to this work.

* Corresponding author

Table S1. Primer sequences

| Gene | Forward primer (5'-3') | | Reverse primer (5'-3') |
| --- | --- | --- | --- |
| IL-6  IL-1β | | TCTGCGCAGCTTTAAGGAGT  TGAGCTCGCCAGTGAAATGA | CCCAGTGGACAGGTTTCTGA  CATGGCCACAACAACTGACG |
| TNF-α | | GACGTGGAACTGGCAGAAGAG | TTGGTGGTTTGTGAGTGTGAG |
| NF-κB1 | | TGTGGGGTTTCAGGATAA | TGGATGATGGCTAAGTGT |
| NF-κB2 | | TGAGGTTCGTTTCTATGAG | CGTGAACTGTTTGGAGTC |
| RelB | | CTGCCATTGAGCGTAAGA | CAAATGTCCCTGCTGGTC |
| GAPDH | | AGGGCCCTGACAACTCTTTT | AGGGGTCTACATGGCAACTG |


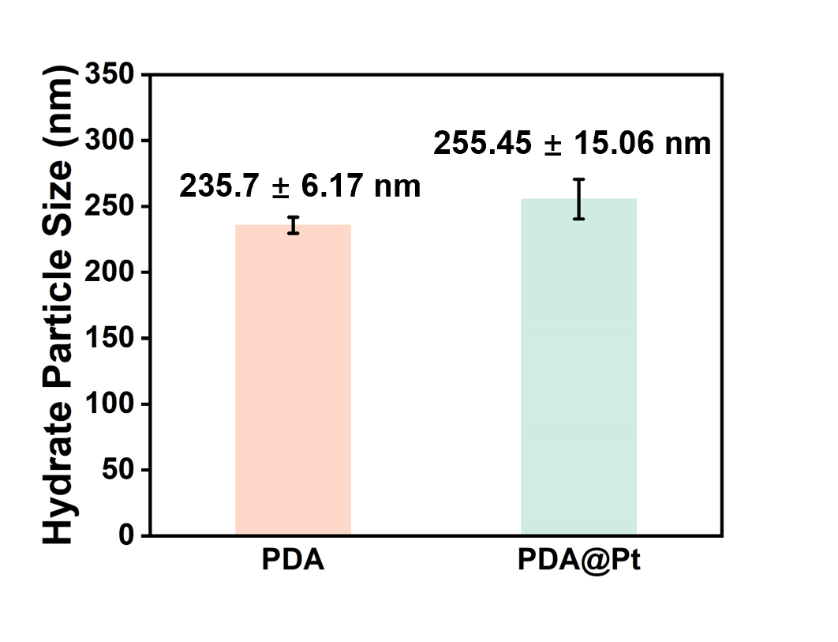


**Figure S1.** The hydrodynamic size of PDA and PDA@Pt is measured by DLS.


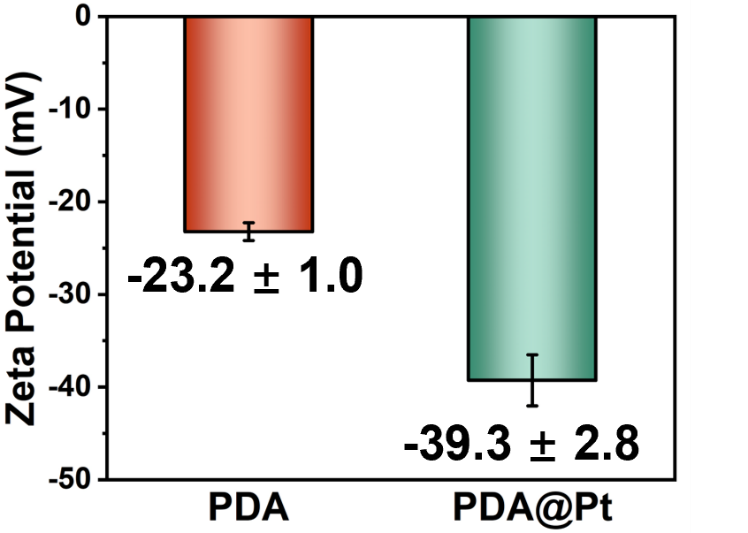


**Figure S2.** The surface zeta potentials of PDA and PDA@Pt.


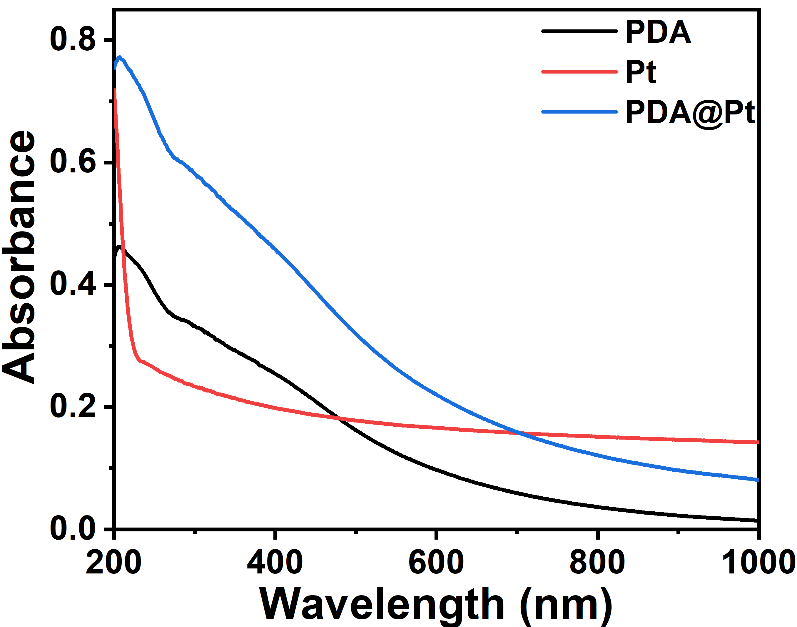


**Figure S3.** The UV-Vis absorption spectra of nano Pt, PDA and PDA@Pt.


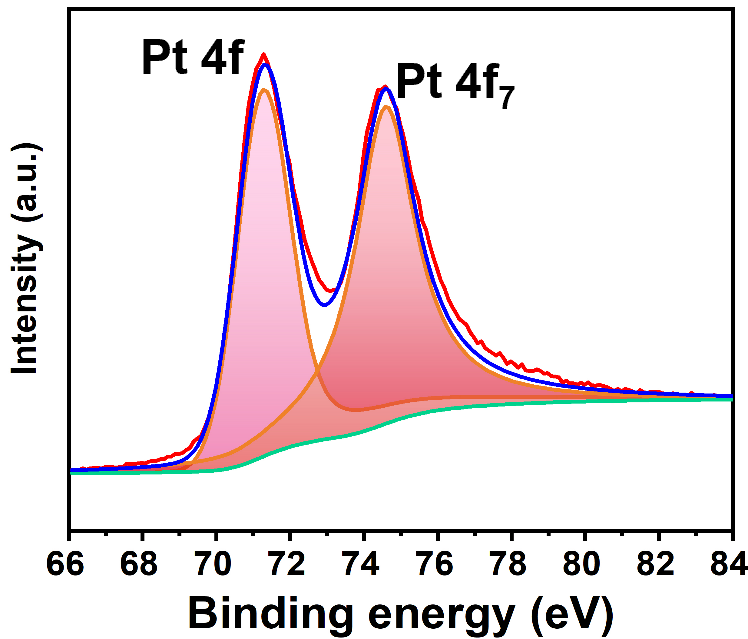


**Figure S4.** The XPS spectra for the element Pt in PDA@Pt.


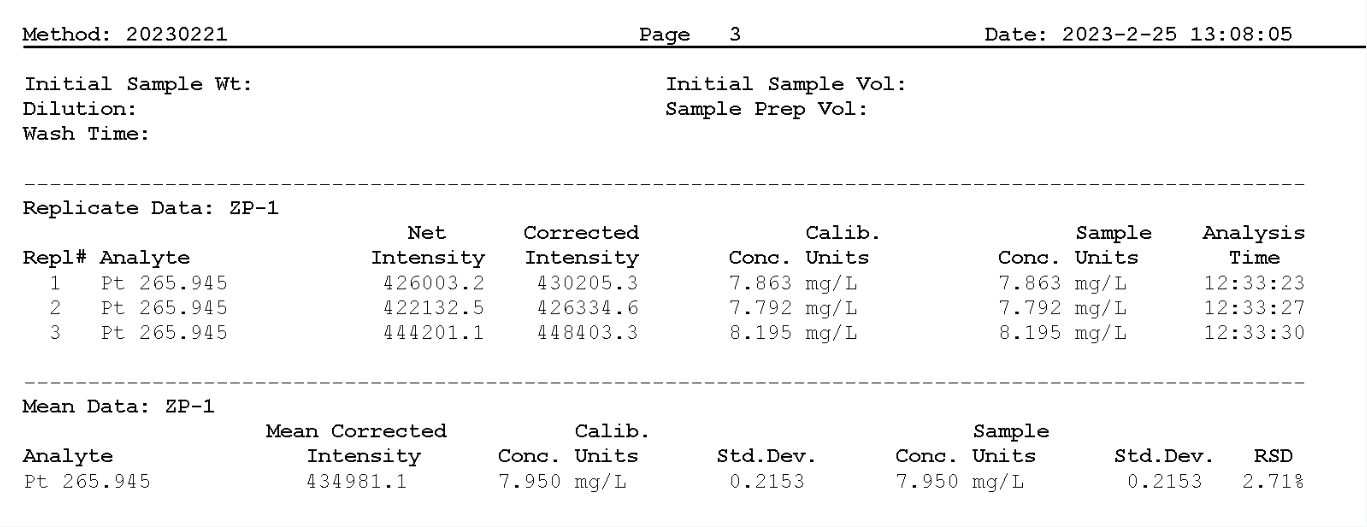


**Figure S5.** The ICP-MS raw data of Pt in PDA@Pt.


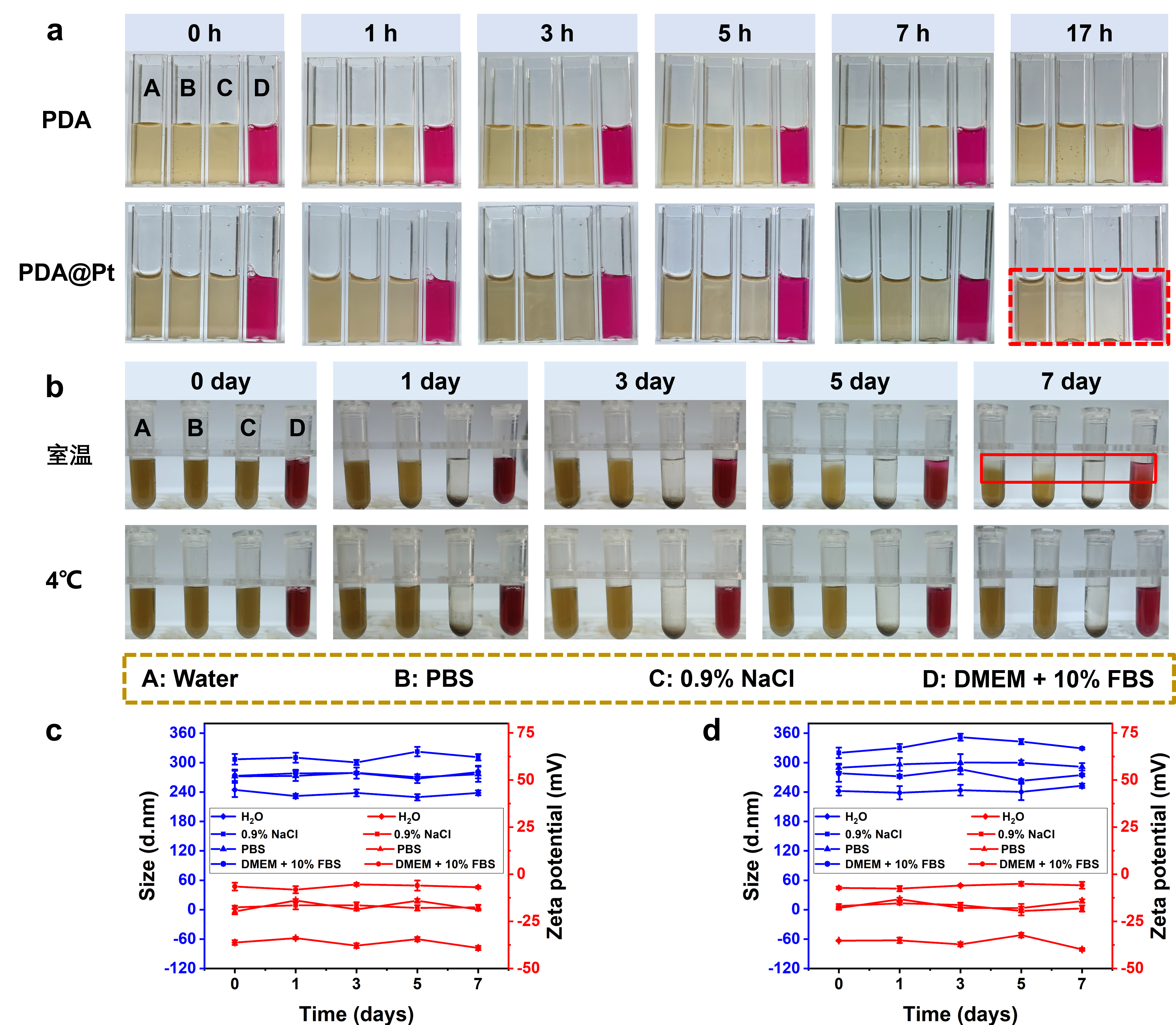


**Figure S6.** (a) The dispersibility of PDA and PDA@Pt in water, PBS, 0.9% NaCl, and DMEM + 10% FBS for 17 h . (b) The dispersibility of PDA@Pt in water, PBS, 0.9% NaCl, and DMEM + 10% FBS for 7 day . The hydrodynamic diameters and zeta potential of PDA@Pt were measured following incubation with water, PBS, 0.9% NaCl, and DMEM + 10% FBS at 4 ℃ (c) and room temperature (d).


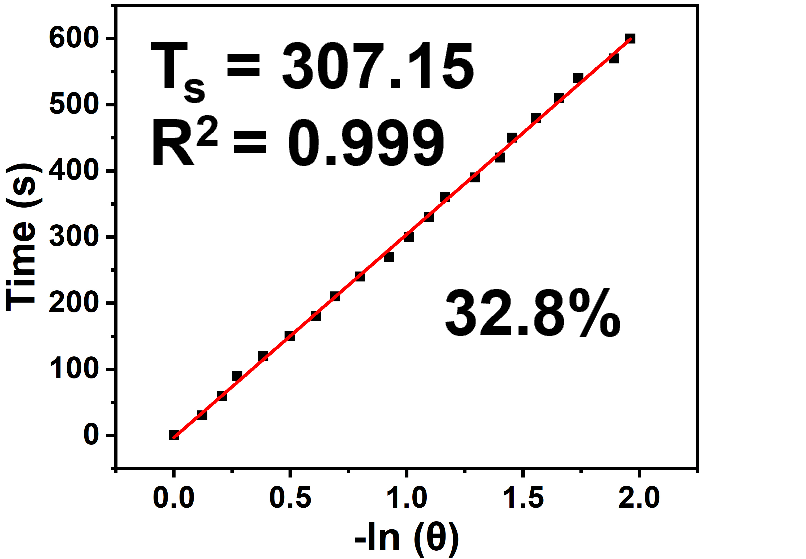


**Figure S7.** The plot of cooling time *vs* −ln θ for PDA@Pt.


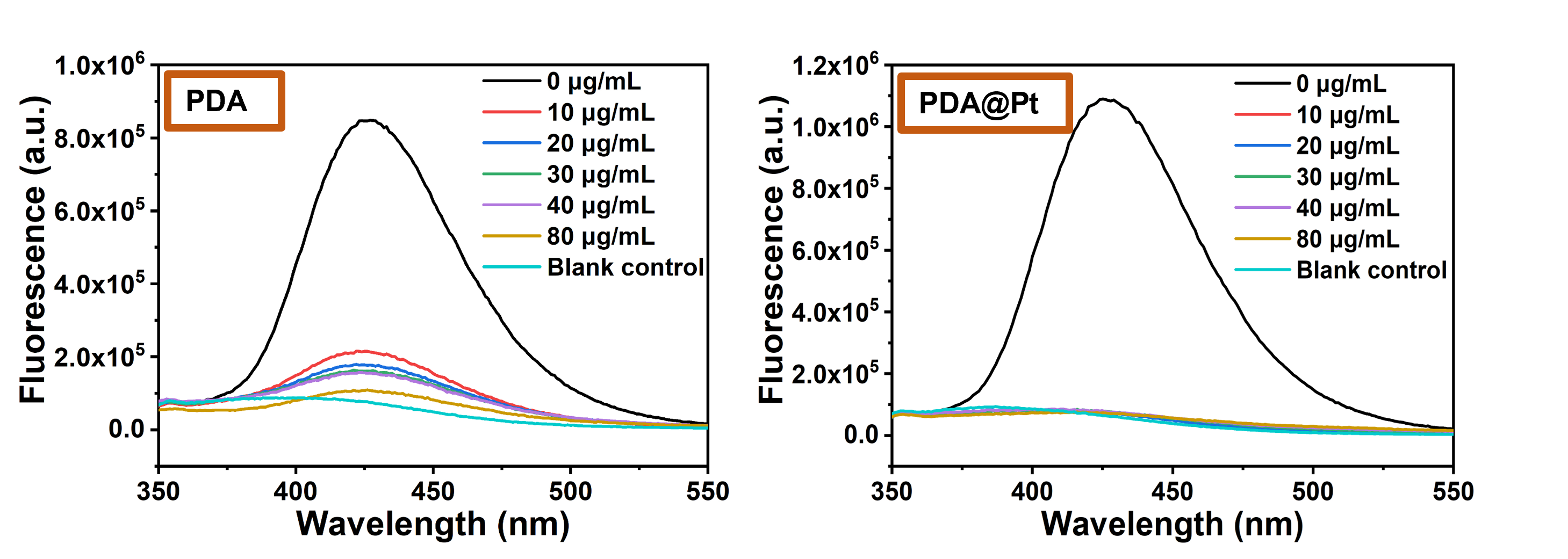


**Figure S8.** The ^•^OH elimination capability of PDA and PDA@Pt. After co-incubating with different concentrations of PDA or PDA@Pt with H_2_O_2_ (10 mM) and TA (5 mM) for 12 h, the fluorescence spectra of TA-OH were obtained. (Ex: 315 nm, Em: 425 nm)


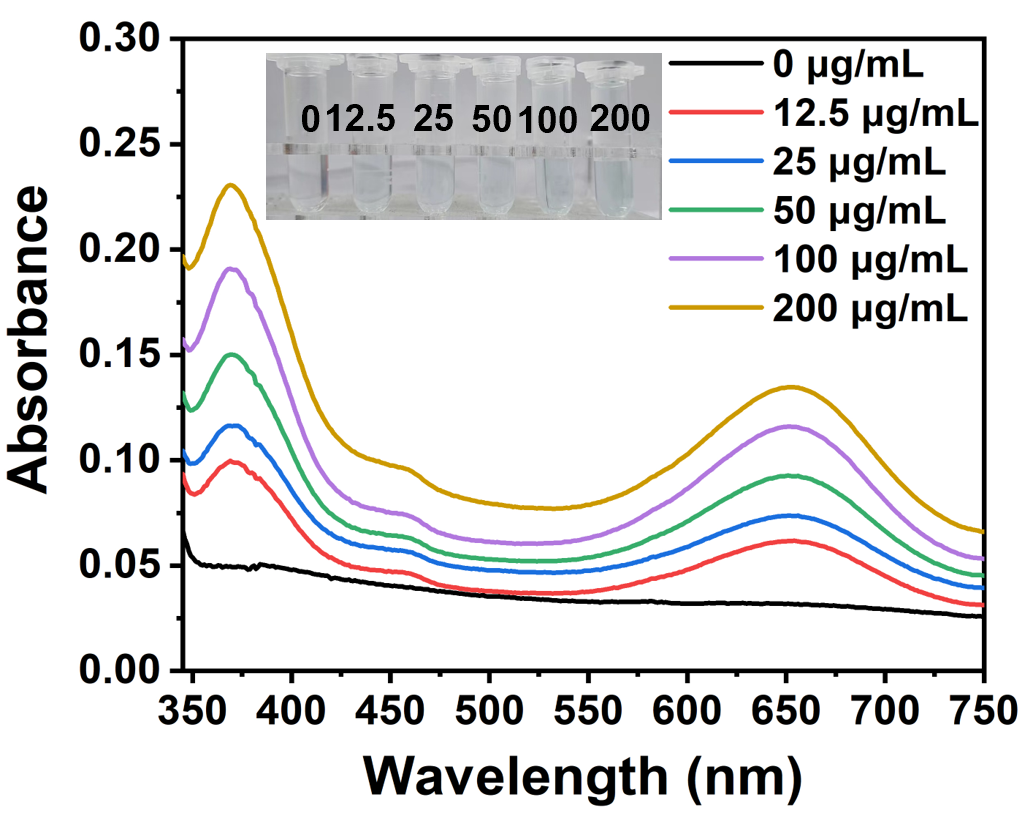


**Figure S9.** The ^•^OH generation of PDA@Pt with graded concentrations (0, 12.5, 25, 50, 100, 200 μg/mL).


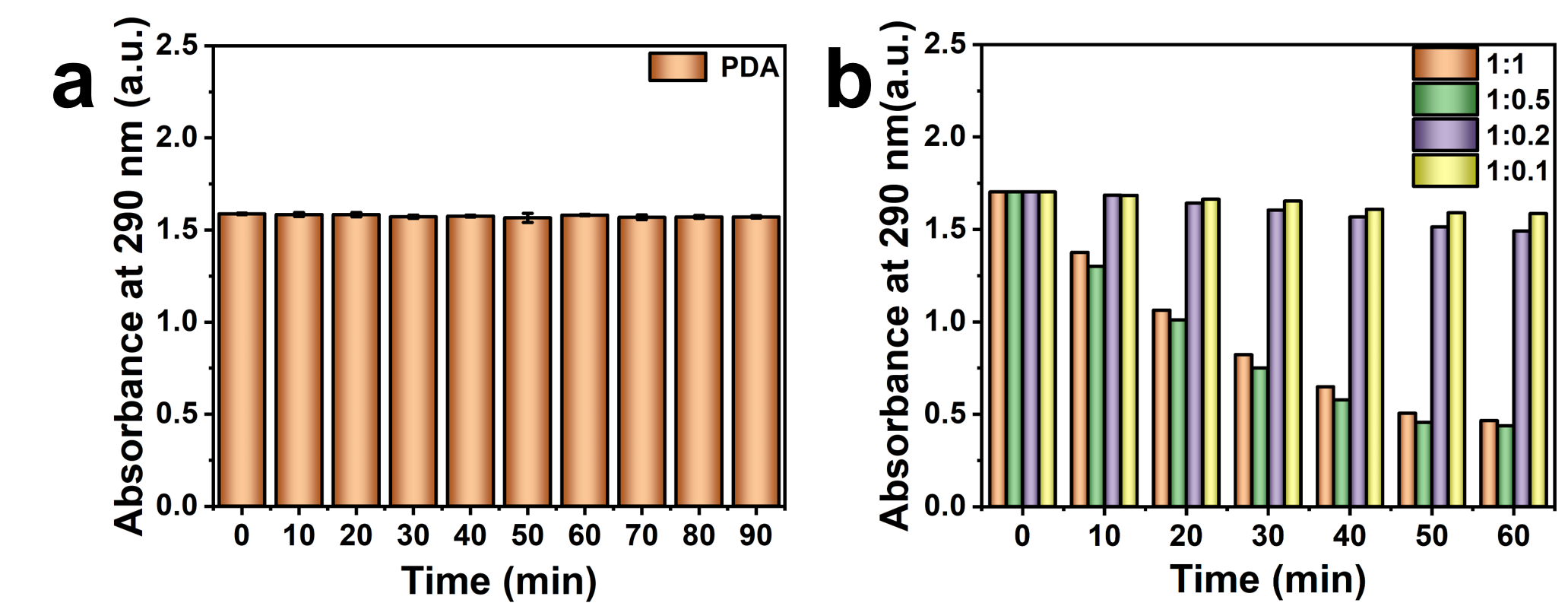


**Figure S10.** a) The time evolution of absorbance at 290 nm every 10 min for monitoring the catalytic degradation of UA in the present of 25 μg/mL of PDA. 100 μM of UA was used in the test. b) After co-incubation of PDA@Pt with UA (100 μM) at different mass ratios (1:1, 1:0.5, 1:0.2, 1:0.1), there is a change in the absorbance of UA (290 nm).


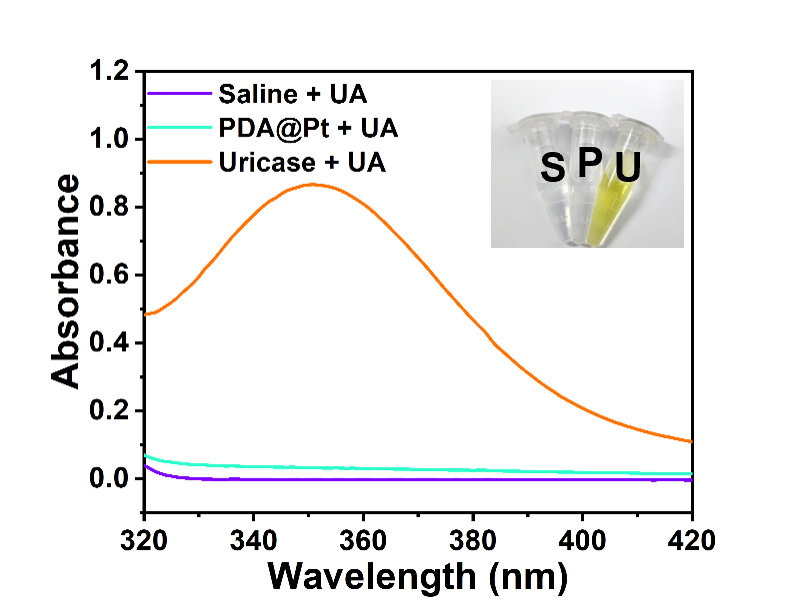


**Figure S11.** The UV-Vis spectrum of I_3_^‑^ is produced by the reaction of KI and H_2_O_2_ during the degradation of UA by different components (Saline, PDA@Pt, and uricase). (UA: 1 mM, PDA@Pt: 50 μg/mL, Uricase: 5 U/mL)


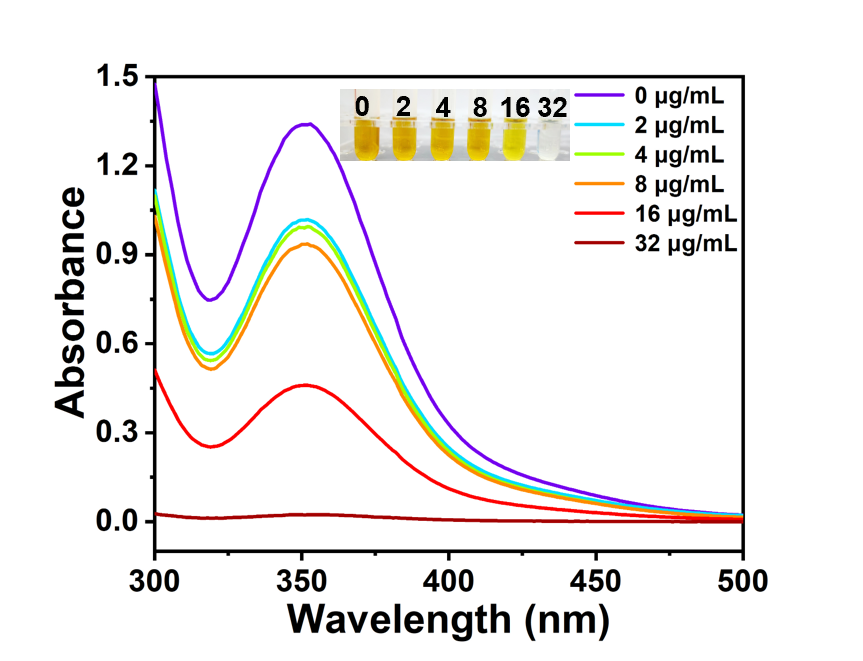


**Figure S12.** The UV-Vis spectrum of I_3_^‑^, a product of the response of H_2_O_2_ and KI, was incubated with different concentrations of PDA@Pt.


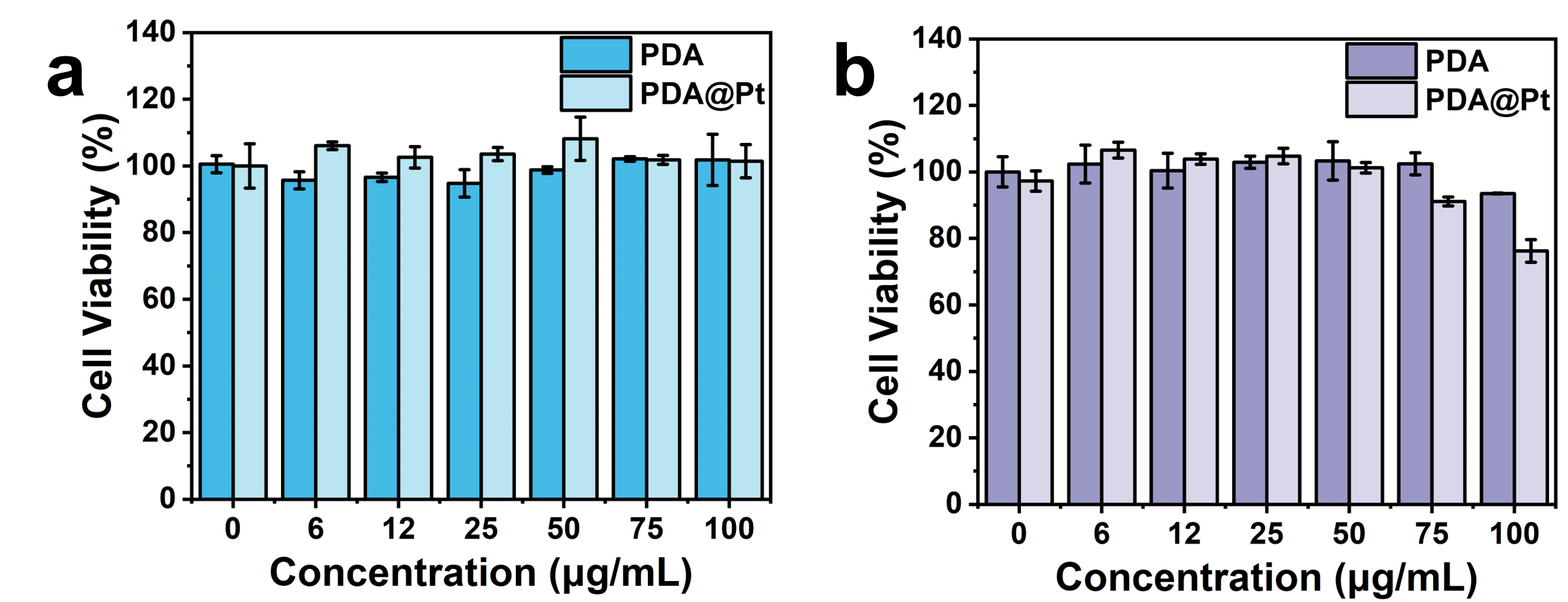


**Figure S13. a)** The cell viability of HUVEC and b) FLS treated with different concentrations (0, 6, 12, 25, 50, 75, and 100 μg/mL) of PDA and PDA@Pt.


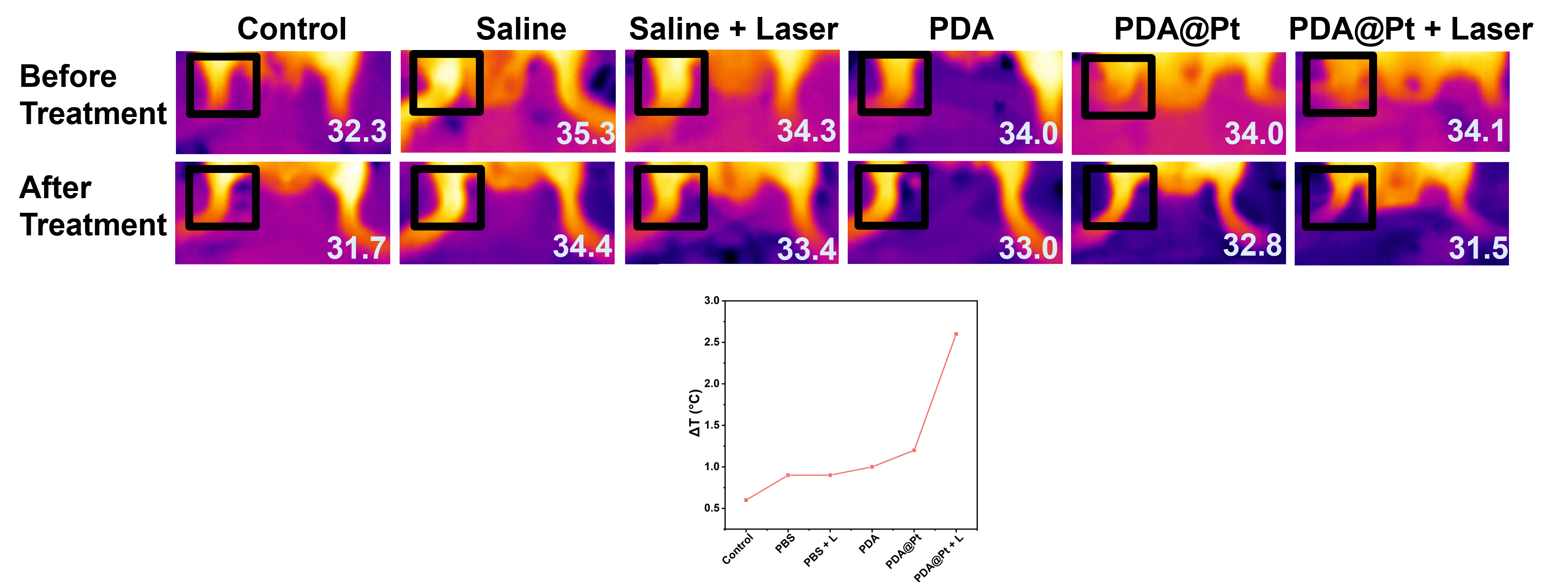
**Figure S14.** The temperature changes in ankle joints before and after treatment in rats with acute gout.

**
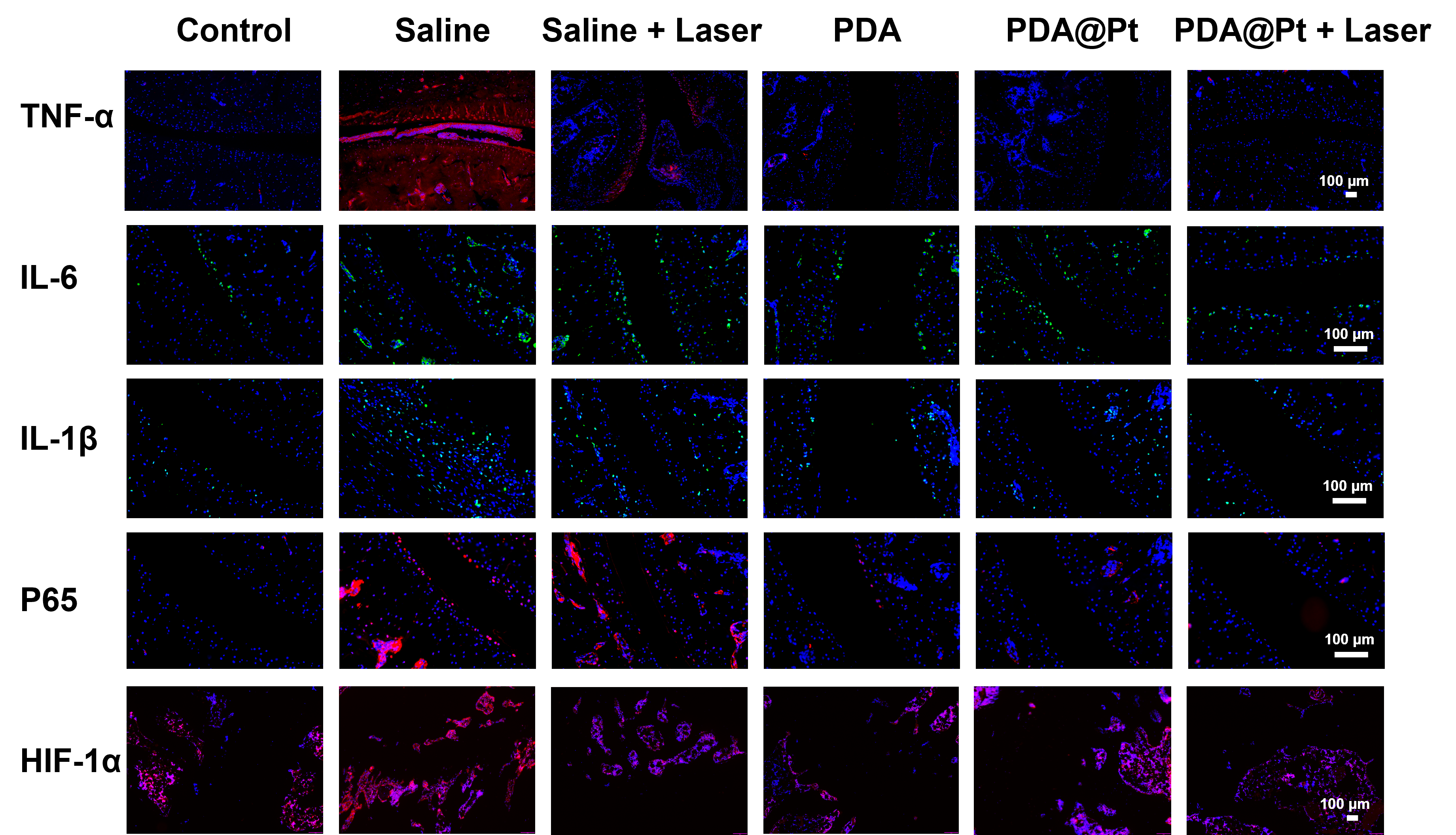
Figure S15.** The immunofluorescent staining of inflammatory factors in the ankle cavity: IL-6, IL-1β, TNF-α, HIF-1α, and P65 (Scale bar: 100 μm).
